# Antibacterial Cyclic Tripeptides from Antarctica-Sponge-Derived Fungus *Aspergillus insulicola* HDN151418

**DOI:** 10.3390/md18110532

**Published:** 2020-10-26

**Authors:** Chunxiao Sun, Ziping Zhang, Zilin Ren, Liu Yu, Huan Zhou, Yaxin Han, Mudassir Shah, Qian Che, Guojian Zhang, Dehai Li, Tianjiao Zhu

**Affiliations:** 1 Key Laboratory of Marine Drugs, Chinese Ministry of Education, School of Medicine and Pharmacy, Ocean University of China, Qingdao 266003, China; sunchunxiao93@163.com (C.S.); zhangziping1998@163.com (Z.Z.); rzl17854263950@163.com (Z.R.); yuliu906@163.com (L.Y.); zhouhuanchn@icloud.com (H.Z.); 17669475579@163.com (Y.H.); s84mudassir@gmail.com (M.S.); cheqian064@ouc.edu.cn (Q.C.); zhangguojian@ouc.edu.cn (G.Z.); 2 Laboratory for Marine Drugs and Bioproducts, Pilot National Laboratory for Marine Science and Technology, Qingdao 266237, China; 3 Open Studio for Druggability Research of Marine Natural Products, Pilot National Laboratory for Marine Science and Technology, Qingdao 266237, China

**Keywords:** cyclic tripeptides, antibacterial, Antarctica sponge-derived fungus, *Aspergillus insulicola*

## Abstract

Three new aspochracin-type cyclic tripeptides, sclerotiotides M–O (**1**–**3**), together with three known analogues, sclerotiotide L (**4**), sclerotiotide F (**5**), and sclerotiotide B (**6**), were obtained from the ethyl acetate extract of the fungus *Aspergillus insulicola* HDN151418, which was isolated from an unidentified Antarctica sponge. Spectroscopic and chemical approaches were used to elucidate their structures. The absolute configuration of the side chain in compound **4** was elucidated for the first time. Compounds **1** and **2** showed broad antimicrobial activity against a panel of pathogenic strains, including *Bacillus cereus*, *Proteus species*, *Mycobacterium phlei*, *Bacillus subtilis*, *Vibrio parahemolyticus*, *Edwardsiella tarda*, MRCNS, and MRSA, with MIC values ranging from 1.56 to 25.0 µM.

## 1. Introduction

Marine life is radically different from its terrestrial counterpart, resulting in an interesting difference between its metabolites [1,2]. Sponge-derived fungi isolated from the marine environment have shown great potential of producing diverse bioactive secondary metabolites [1,2,3,4]. Cyclic peptides are a class of essential metabolites that are widely present in marine tunicates [5], sponge [6], algae [7], bacteria [8], fungi [9,10], etc. The structure of aspochracin-type cyclic tripeptides usually contains a unique macro-cyclic ring and a polyketide side chain. For the macro-cyclic ring, the most common features are a 12-member (composed of Ala-Val-Orn) and 13-member (composed of Ala-Val-Lys) ring. Only 15 of the aspochracin-type cyclic tripeptides have been obtained from natural sources (Figure S1). Their structures were mainly different regarding the polyketide side chains, the constitution of amino acids, and the *N*-methylation level in the amino acid moieties. Those chemistry diversities were able to be generated by chemical transformations. For example, some analogs can be synthesized from JBIR-15 or aspochracin via photoisomerization-initiated radical reaction or air oxidation during the fermentation or subsequent isolation steps [10]. Some of the analogs exhibited antifungal or anti-inflammatory activities [10,11,12,13].

**Figure 1:**
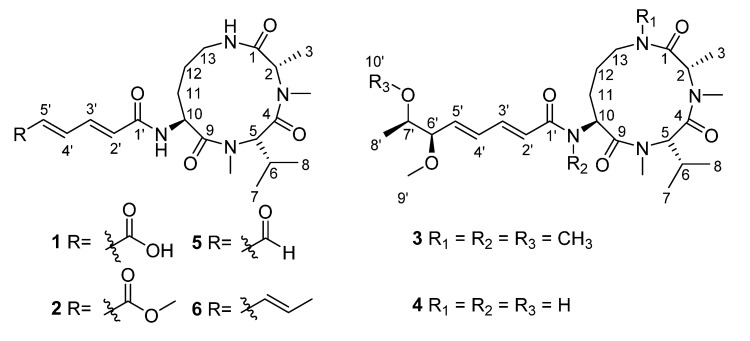
Structures of compounds **1**–**6**.

During our ongoing research on bioactive natural products from Antarctic marine-derived fungi [14,15,16,17], *Aspergillus insulicola* HDN151418, a fungal strain isolated from an unidentified sponge, was chosen based on its unique HPLC-UV profile (series UV absorption around 260 nm) (Figure S2) and its antibacterial activity against MRCNS and MRSA (detected by the paper diffusion method). Consequently, chemical investigation resulted in the identification of three new aspochracin-type cyclic tripeptides, sclerotiotides M–O (**1**–**3**), together with three known compounds, sclerotiotide L (**4**), sclerotiotide F (**5**), and sclerotiotide B (**6**) (Figure 1). Compounds **1** and **2** represent the first example of an aspochracin-type cyclic tripeptide that possess a hexa-2,4-dienedioic acid/methyl ester moiety. Here, we address the isolation, elucidation of the structure, and biological activities of the new aspochracin-type cyclic tripeptides, sclerotiotides M–O (**1**–**3**).

## 2. Results and Discussion

The fungus *A. insulicola* HDN151418 was cultured for 30 days under static condition (30 L). The crude extract (32.3 g) was fractionated and purified by LH-20, ODS, and HPLC, sequentially, yielding compounds **1** (10.2 mg), **2** (5.7 mg), **3** (6.5 mg), **4** (6.5 mg), **5** (20.2 mg), and **6** (12.0 mg).

**Figure 2:**
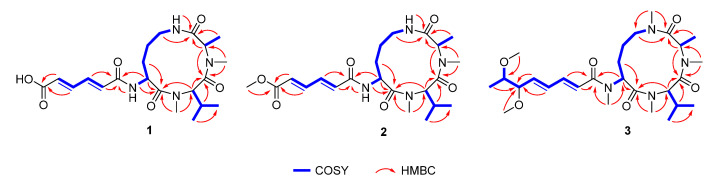
Key 2D NMR correlations of **1**–**3**.

**Figure 3:**
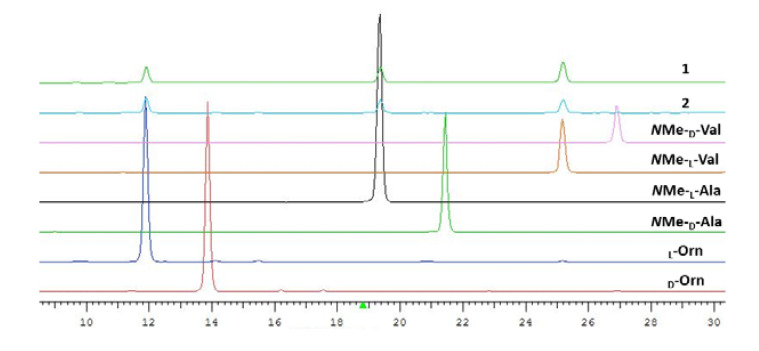
HPLC analysis of the FDAA derivatives of the compounds **1** and **2** and the standard amino acids.

Sclerotiotide M (**1**) was isolated as a pale yellow amorphous powder. The molecular formula was assigned as C_21_H_32_N_4_O_6_ based on the HRESIMS ion peak at *m/z* 435.2246 [M − H]^−^ (calcd for C_21_H_31_N_4_O_6_, 435.2249). The IR spectrum showed absorption bands for amide groups at 3394 cm^−1^ and 1681 cm^−1^. The ^1^H and ^13^C NMR spectra of **1** showed two amide NH protons (*δ*_H_ 7.47 and 8.39), two *N*-methyl protons (*δ*_H_ 2.83), and three characteristic *α*-methine signals (*δ*_H_ 4.50, 4.71, and 4.97). These features are characteristic of a tripeptide structure. Comprehensive analysis of the 1D NMR data of **1** (Table 1 and Table 2) revealed that it is very similar to those of sclerotiotide F (**5**) [10]. The only difference between **1** and **5** was the presence of a carboxyl acid group (*δ*_H/C_ 12.5 brs/167.5) in **1** instead of an aldehyde group in **5**. The hexa-2,4-dienedioic acid side chain of **1** was further confirmed by the COSY correlations of H-2′/H-3′/H-4′/H-5′ and HMBC correlations from H-2′ to C-1′ and H-5′ to C-6′ (Figure 2). The geometric configurations of the two double bonds in the side chain are assigned as *E* on the basis of the large coupling constants (*J*_2′-3′_ = 14.3 Hz, *J*_4′-5′_ =14.4 Hz) and ROEs of H-2′/H-4′ and H-3′/H-5′. The absolute configurations of the α-carbons in the three amino acid units were determined by Marfey′s method [18,19]. In detail, sclerotiotide M (**1**) was hydrolyzed into free amino acids, which were further derivatized with FDAA (1-fluoro-2-4-dinitrophenyl-5-l-alanine amide). HPLC analyses of FDAA derivatives of the hydrolysates and authentic samples revealed that the amino acid residues in **1** were l-*N*Me-Val, l-*N*Me-Ala, and l-Orn (Figure 3). Thus, sclerotiotide M (**1**) was established as (2′*E*,4′*E*)-*cyclo*-[(*N*Me-l-Ala) -(*N*Me-l-Val)-(*N*_α_-5-carboxyhexa-2,4-dienoyl-l-Orn)].

Sclerotiotide N (**2**) was obtained as a pale yellow amorphous powder. The HRESIMS peak at *m/z* 451.2556 [M + H]^+^ indicated that its molecular formula was C_22_H_34_N_4_O_6_, which is 14 Da more than that of compound 1. The NMR data of **2** (Table 1 and Table 2) were almost the same as those of **1** except for the additional signal of a methoxy group (*δ*_H_ 3.69, s). The methoxy group was determined to be linked to C-6′ by the HMBC correlation between H-7′ and C-6′ (Figure 2). The geometric configurations of the two double bonds in the side chain are both assigned as *E* on the basis of the coupling constants (*J*_2′-3′_ = 14.6 Hz, *J*_4′-5′_ =14.8 Hz) and ROEs of H-2′/H-4′ and H-3′/H-5′. Marfey′s analysis was used to determine the absolute configuration of the amino acids present in the cyclic tripeptide [18]. The absolute configurations of the amino acid units of 2 were determined to be identical to 1. Acid hydrolysis and FDAA derivatization revealed l-*N*Me-Ala, l-*N*Me-Val, and l-Orn by HPLC analysis (Figure 3). Thus, sclerotiotide N (**2**) was established as (2′*E*,4′*E*)-*cyclo*-[(*N*Me-l-Ala)-(*N*Me-l-Val)-(*N*_α_-6-methoxy-6-oxohexa-2,4-dienoyl-l-Orn)].

**Figure 4:**
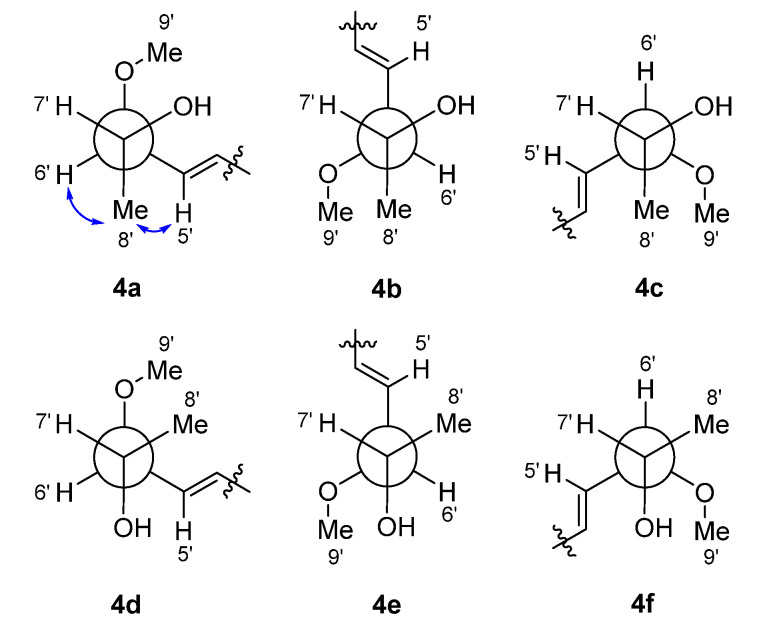
Newman projections for C-6′/C-7′ of **4**. All possible relative conformations are shown: 6′R*,7′R* (**4a**–**4c**) and 6′S*, 7′R* (**4d**–**4f**). Observed ROESY correlations are presented as arrowed line.

**Figure 5:**
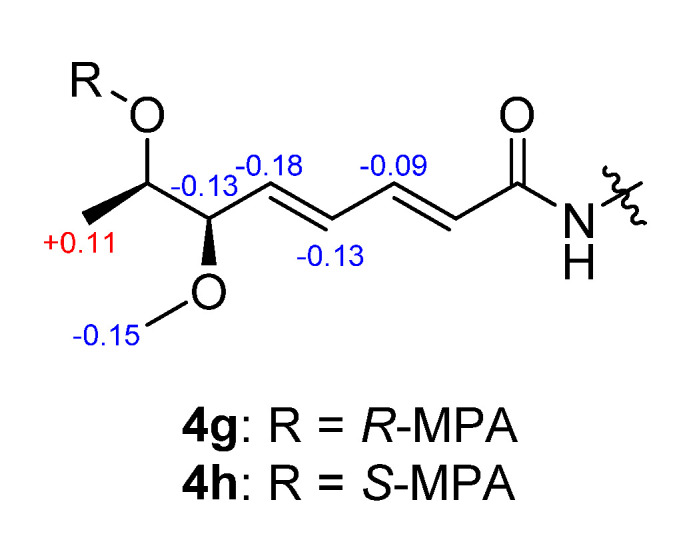
Δ*δ^RS^* values of **4g** and **4h**.

Sclerotiotide L (**4**) and sclerotiotide O (**3**) were isolated as pale yellow powders with the molecular formulas of C_24_H_40_N_4_O_6_ and C_27_H_46_N_4_O_6_, respectively, according to the analysis of HRESIMS data. The NMR data of **4** were identical to that of sclerotiotide L [12], indicating that they share the same planar structure (Table S1). Sclerotiotide L was first reported in 2018, while the stereochemistry of C-6′ and C-7′ remain unknown. Here, the absolute configuration of them was first determined using coupling constants analysis and Mosher′s method. The small coupling constant (^3^*J*_H-6′, H-7′_ = 4.9 Hz) between H-6′ and H-7′ indicates they are in a *gauche* conformation, which allowed focusing on two (**4a** and **4e**) of the six possible relative conformations (Figure 4). The relative configuration was further determined to be 6′R* and 7′R* by the ROESY correlations of H-6′/H-8′/H-5′ (Figure 4, **4a**). The absolute configuration of C-7′ was determined by Mosher′s method [20]. Accordingly, compound **4** was derivatized into the esters **4g** and **4h** with (*R*)- and (*S*)-MPA (*α*-methoxyphenylacetic acid), respectively. The chemical shifts differences Δ*δ^RS^* suggested *R* configuration at C-7′ (Figure 5). Thus, compound **4** was established as (2′*E*,4′*E*)-*cyclo*-[(*N*Me-l-Ala)-(*N*Me-l-Val)-(*N*_α_-(6*R*,7*R*)-7-hydroxy-6-methoxyocta-2,4-dienoyl-l-Orn)]. Distinguished from **4**, compound **3** possessed three extra methyls (*δ*_H_ 2.83, s; 3.08, s; 3.25, s), which were assigned at N_Orn (*α*)_, N_Orn (*ω*)_, and 7′-OH on the basis of HMBC correlations from N_Orn (*α*)_-CH_3_ to C-1 and C-13, from N_Orn (*ω*)_-CH_3_ to C-9 and C-11, and from H-10′ to C-7′, respectively (Figure 2). The geometric configurations of the two double bonds in the side chain are both assigned as *E* on the basis of the coupling constants (*J*_2′-3′_ = 15.0 Hz, *J*_4′-5′_ =15.5 Hz) and ROEs of H-2′/H-4′ and H-3′/H-5′. Finally, the absolute configurations of C-6′ and C-7′ in **3** were assigned to be the same as **4** by the semisynthesis of **3** from **4**. Compound **4** was treated with sodium hydride in tetrahydrofuran to obtain **4A**. The identical NMR chemical shifts, ECD curves, and specific rotation values between **4A** and **3** indicated that **3** displayed the same stereochemistry with **4** (Figure S40). Thus, compound **3** was established as (2′*E*,4′*E*)-*cyclo*-[(*N*Me-l-Ala)-(*N*Me-l-Val)-(*N*_α_Me-(6*R*,7*R*)-6,7-dimethoxyocta-2,4-dienoyl-l-Orn)].

It is well-known that some artificial compounds are formed from natural compounds due to oxidation when exposed to air [10]. In order to verify the origin of compounds **1**–**6**, the fermentation broth of *A. insulicola* HDN151418 was dried under a freeze dryer and extracted by MeCN and further analyzed by LC-MS. Only compounds **1**, **2**, **5**, and **6** were detected. After compound **6** was dissolved in solvent mixture MeOH-H_2_O and exposed to air for two weeks, compounds **4** and **5** were also detected from the product, indicating that compounds **4** and **5** could be formed from **6** during fermentation or isolation steps. The methoxy groups in **3** and **4** could come from methanol during isolation steps.

Compounds **1**–**6** were tested for their cytotoxic activities on 16 cancer cell lines (K562, BEL-7402, HCT-116, A549, Hela, L-02, GES-1, U87, ASPC-1, SH-SY5Y, PC-3, MGC-803, HO8910, MCF-7, MDA-MB-231, and NCI-H446); none of them showed activity (IC_50_ > 30 *μ*M). The antimicrobial activities were tested against eight pathogenic strains, including *Bacillus cereus*, *Proteus species*, *Mycobacterium phlei*, *Bacillus subtilis*, *Vibrio parahemolyticus*, *Edwardsiella tarda*, MRCNS, and MRSA. Compounds **1** and **2** showed broad inhibition against a panel of strains with MIC values ranging from 1.56 to 25.0 μM, while compound **3**–**6** were less active (Table 3), which indicated that the carboxyl group or its methyl ester display an important role for antibacterial activities. Notably, **1** and **2** showed potent activity against *M. phlei*, which provide potential candidates for antitubercular drug development. Additionally, no cytotoxicities further expands their pharmacological potential.

## 3. Materials and Methods

### 3.1. General Experimental Procedures

By means of a JASCO P-1020 digital polarimeter developed by JASCO Corporation, Tokyo, Japan, optical rotations for all new compounds were calculated in methanol. Nuclear magnetic resonance data were obtained on a Bruker AVANCE NEO 400 MHz spectrometer made by Bruker Corporation, Karlsruhe, Germany, and an Agilent 500 MHz DD2 spectrometer by Agilent Technologies Inc., Santa Clara, CA, USA and a JEOL JNM-ECP600 spectrometer by JEOL, Tokyo, Japan using TMS as an internal standard. The ECD spectrum was measured on a JASCO J-815 spectropolarimeter made by JASCO Corporation, Tokyo, Japan. By using KBr discs in the Bruker Tensor-27 spectrophotometer made by Bruker Corporation, Karlsruhe, Germany, IR data were collected. In addition, HRESIMS data were recorded on a LTQ Orbitrap XL mass spectrometer made by Thermo Fisher Scientific, Waltham, MA, USA. UV spectra were carried out on Waters 2487 developed by Waters Corporation, Milford, MA, USA. Column chromatography was performed using the following chromatographic substrates: silica gel (300−400 mesh; Qingdao Marine Chemical Industrials, Qingdao, China), Sephadex LH-20 (developed by Amersham Biosciences, San Francisco, CA, USA). The compounds were purified by HPLC made by the Waters company equipped with a 2998 PDA detector and a C18 column (YMC-Pack ODS-A, 10 × 250 mm, 5 μm, 3 mL/min). LC-MS was recorded in ESI mode on an Acquity UPLC H-Class connected to a SQ Detector 2 mass spectrometer using a BEH C18 column (1.7 µm, 2.1 × 50 mm, 1 mLperminute) constructed by Waters Corporation, Milford, CT, USA.

### 3.2. Fungal Material and Fermentation

*Aspergillus insulicola* HDN151418 was isolated from an unidentified sponge sample collected 410 m deep from Prydz Bay, Antarctica at a latitude and longitude of E 68.7°, S 67.2° while identified as *Aspergillus insulicola* based on internal transcribed spacer DNA sequencing. The sequence is available with the accession number MT898544 at Genbank and has been submitted to the Key Laboratory of Marine Drugs working under the Ministry of Education of China, School of Medicine and Pharmacy, Ocean University of China.

To prepare the seed culture, the strain was cultured on potato dextrose agar (PDA) at 28 °C for 7 days and then was transferred to 30 mL potato dextrose broth (PDB) medium in a 100 mL flask. After fermentation for 3 days on a rotary shaker at 180 rpm at 28 °C, 1 mL aliquot of the liquid culture was transferred to 300 mL of PDB medium in a 1000 mL flask for scale-up. The culture was incubated in static condition for 30 days before extraction.

### 3.3. Isolation and Purification of the Compounds

The total fermentation broth (30 L) was harvested and the supernatant was separated from the mycelia by using a filter cloth. The solvent-associated extraction was performed, the supernatant was extracted with EtOAc (3 × 30 L), and the mycelia was crushed into small pieces by using an electric cutter and macerated with MeOH (3 × 15 L). Based on the corresponding HPLC and TLC profiles, both extracts were combined, and the subsequent removal of solvent afforded 32.3 g of reddish-brown crude extract. Moreover, the extract was fractioned by using vacuum chromatography on silica gel followed by stepped gradient elution via DCM-MeOH (10:0 to 0:10) solvent combination to obtain ten subfractions (Fr.1 to Fr.10). Then, Fr.3 was separated by an ODS column by using MeOH andH_2_O in the form of a stepped gradient, 20:80 to 50:50 to obtain six subfractions (Fr.3-1 to Fr.3-6). Fr.3-3 was further subjected to a Sephadex LH-20 column and eluted with MeOH to provide four subfractions (Fr.3-3-1 to Fr.3-3-4). Fr.3-3-2 was purified by HPLC eluted with MeOH-H_2_O (30:70) to obtain compounds **1** (10.2 mg, *t*_R_ = 15 min) and **5** (20.2 mg, *t*_R_ = 18 min). Likewise, Fr.5 was divided into three subfractions (Fr5-1 to Fr.5-3) by MPLC using a stepped gradient elution of MeOH-H_2_O (40:60 to 60:40). Fr.5-2 was separated by HPLC eluted with MeCN-H_2_O (30:70) to obtain compounds **2** (5.7 mg, *t*_R_ = 23 min) and **4** (6.5 mg, *t*_R_ = 25 min). With the same procedure as used for fraction Fr.5, Fr.8 was purified by HPLC eluted with MeCN-H_2_O (70:30) to yield compounds **3** (6.5 mg, *t*_R_ = 31 min) and **6** (12.0 mg, *t*_R_ = 33 min), respectively.

**Sclerotiotide M (1)**: pale yellow, amorphous powder; [α]${}_{D}^{20}$−66 (*c* 0.5, MeOH); UV (MeOH) *λ*_max_ (log *ε*) 206 (4.00), 270 (4.40) nm; IR *ν*_max_ 3394, 2937, 1681, 1527, 1205, 1135, 838, 584 cm^−1^; ^1^H and ^13^C NMR data, Table 1 and Table 2; HRESIMS *m/z* 435.2246 [M − H]^−^ (calcd for C_21_H_31_N_4_O_6_ 435.2249).

**Sclerotiotide N (2)**: pale yellow, amorphous powder; [α]${}_{D}^{20}$−72 (*c* 0.2, MeOH); UV (MeOH) *λ*_max_ (log *ε*) 206 (4.05), 272 (4.35) nm; IR *ν*_max_ 3384, 2939, 1673, 1636, 1540, 1456, 1205, 1137, 1027, 839, 580 cm^−1^; ^1^H and ^13^C NMR data, Table 1 and Table 2; HRESIMS *m/z* 451.2556 [M + H]^+^ (calcd for C_22_H_35_N_4_O_6_ 451.2551).

**Sclerotiotide O (3)**: pale yellow, amorphous powder; [α]${}_{D}^{20}$−59 (*c* 0.3, MeOH); UV (MeOH) *λ*_max_ (log *ε*) 206 (3.90), 258 (4.50) nm; IR *ν*_max_ 3336, 2931, 1679, 1534, 1453, 1206, 1138, 1027, 840, 801, 722 cm^−1^; ^1^H and ^13^C NMR data, Table 1 and Table 2; HRESIMS *m/z* 523.3484 [M + H]^+^ (calcd for C_27_H_47_N_4_O_6_ 523.3490).

**Sclerotiotide L (4)**: pale yellow, amorphous powder; [α]${}_{D}^{20}$−52 (*c* 0.1, MeOH); UV (MeOH) *λ*_max_ (log *ε*) 206 (3.90), 258 (4.50) nm; IR *ν*_max_ 3384, 2972, 2265,1684, 1450, 1204, 1139, 1029, 833, 800, 721 cm^−1^; ^1^H and ^13^C NMR data, Table 1 and Table 2; HRESIMS *m/z* 481.3017 [M + H]^+^ (calcd for C_24_H_41_N_4_O_6_ 481.3021).

### 3.4. Absolute Configuration Assignments of Sclerotiotides M–N (**1**–**2**).

Compounds **1** and **2** (1.5 mg each) were reacted with 6 N HCl (1.5 mL) at 110 ℃ for 15 h. The solution was dried and separately dissolved in H_2_O (100 µL). Then, 0.50 µM of FDAA (1-fluoro-2-4-dinitrophenyl-5-l-alanine amide) was added to 100 µL of acetone, and 1 N NaHCO_3_ (50 µL) to form a mixture. The mixtures were heated for 2 h at 43 ℃ and then quenched by the addition of 2 N HCl (100 µL). Amino acid standards were derivatized with FDAA in a similar way. The resulting FDAA derivatives of compound **1**, compound **2**, l- and d-*N*Me-Ala, l- and d-*N*Me-Val, and l- and d-Orn were analyzed by HPLC eluted with a linear gradient of MeCN (A) and 0.10% aqueous TFA (B) from 50% to 100% in an over 30 min with UV detection at 320 nm. The measured retention times of amino acid standards are as follows (in min): 21.44 for d-*N*Me-Ala-FDAA, 19.36 for l-*N*Me-Ala-FDAA, 26.88 for d-*N*Me-Val-FDAA, 25.17 for l-*N*Me-Val-FDAA, 13.87 for d-Orn-FDAA, and 11.89 for l-Orn-FDAA. Retention times for the FDAA derivatives of **1** and **2** are as follows (in min): 25.17, 19.36, and 11.89, indicating all the amino acid residues in **1** and **2** are an *S* configuration [18,19].

### 3.5. Assay of Cytotoxicity Inhibitory Activity

Cytotoxicity of compounds **1**–**6** were screened against the 16 human cancer cell lines as previously reported in which Adriamycin was used as the positive control [9,21]. HeLa (human epithelial carcinoma cell line), HCT-116 (human colon cancer cell line), MCF-7 (human breast adenocarcinoma cell line), A549 (human lung adenocarcinoma cell line), SH-SY5Y (human neuroblastoma cell line), MDA-MB-231 (human breast cancer cell line), HO8910 (human ovarian carcinoma cell line), and MGC-803 (human stomach carcinoma cell line) were ordered from Shanghai Institute of Biochemistry and Cell Biology, Chinese Academy of Sciences (Shanghai, China). K562 (human leukaemia cell line), BEL-7402 (human hepatic carcinoma cell line), L-02 (human normal hepatic cell line), GES-1 (human gastric epithelial cell line), U87 (human primary glioblastoma cell line), ASPC-1 (human pancreatic cancer cell line), PC-3 (human prostate carcinoma cell line), and NCI-H446 (human small cell lung cancer cell line) were ordered from the American Type Culture Collection (ATCC, Gaithersburg, MD, USA).

### 3.6. Assay of Antimicrobial Activity

The antimicrobial activities of **1**–**6** against *Bacillus cereus*, *Proteus species*, *Mycobacterium phlei*, *Bacillus subtilis*, *Vibrio parahemolyticus*, *Edwardsiella tarda*, MRCNS, and MRSA were evaluated as previously reported by using the agar dilution method [22,23]. All experiments were performed in triplicates, and ciprofloxacin was used as a positive control. All strains were donated by the Qingdao municipal hospital.

### 3.7. Preparation of MPA Esters Derived from **4** (**4g** and **4h**)

The sample of compound **4** (0.5 mg each) was reacted with (*R*)- or (*S*)-MPA (5.0 mg) with *N*, *N*′-dicyclohexylcarbodiimide (DCC, 1.0 mg) and 4-dimethylaminopyridine (DMAP, 0.1 mg) in dry CDCl_3_ (0.5 mL). After stirring for 2.0 h at 0 °C, the residue was evaporated under vacuum pressure and purified by HPLC eluted with 60% acetonitrile/H_2_O to give the (*R*)-MPA ester (**4g**) and (*S*)-MPA ester (**4h**).

(*R*)-MPA Ester (**4g**): pale yellow powder; ^1^H NMR (400 MHz, DMSO-*d*_6_) *δ*_H_ 8.23 (d, *J* = 7.7 Hz, 1H), 7.48 (t, *J* = 6.7 Hz, 1H), 7.28-7.38 (m, 5H), 6.91 (dd, *J* = 15.1, 11.1 Hz, 1H), 6.24 (dd, *J* = 15.3, 11.2 Hz, 1H), 6.18 (d, *J* = 14.9 Hz, 1H), 5.71 (dd, *J* = 15.3, 7.5 Hz, 1H), 4.98 (d, *J* = 10.1 Hz, 1H), 4.88 (s, 1H), 4.74 (m, 1H), 4.51 (m, 1H), 4.11 (m, 1H), 3.65 (m, 1H), 3.31 (s, 3H), 3.06 (m, 1H), 3.03 (m, 1H), 2.88 (m, 1H), 2.85 (s, 3H), 2.85 (s, 3H), 2.18 (m, 1H), 1.98 (m, 2H), 1.63 (m, 2H), 1.49 (m, 1H), 1.39 (d, *J* = 7.1 Hz, 3H), 1.13 (d, *J* = 6.5 Hz, 3H), 0.81 (d, *J* = 6.4 Hz, 3H), 0.65 (d, *J* = 6.7 Hz, 3H). HRESIMS *m/z* 627.3387 [M − H]^−^ (calcd for C_33_H_49_N_4_O_8_, 627.3399).

(*S*)-MPA Ester (**4h**): pale yellow powder; ^1^H NMR (400 MHz, DMSO-*d*_6_) *δ*_H_ 8.23 (d, *J* = 7.7 Hz, 1H), 7.50 (t, *J* = 6.7 Hz, 1H), 7.30-7.40 (m, 5H), 7.00 (dd, *J* = 14.9, 11.2 Hz, 1H), 6.37 (dd, *J* = 15.3, 11.2 Hz, 1H), 6.24 (d, *J* = 14.9 Hz, 1H), 5.89 (dd, *J* = 15.3, 7.5 Hz, 1H), 4.99 (d, *J* = 10.1 Hz, 1H), 4.86 (s, 1H), 4.74 (m, 1H), 4.51 (m, 1H), 4.11 (m, 1H), 3.78 (m, 1H), 3.29 (s, 3H), 3.21 (s, 3H), 3.03 (m, 1H), 2.88 (m, 1H), 2.85 (s, 3H), 2.85 (s, 3H), 2.21 (m, 1H), 1.98 (m, 2H), 1.63 (m, 2H), 1.48 (m, 1H), 1.39 (d, *J* = 7.1 Hz, 3H), 1.02 (d, *J* = 6.5 Hz, 3H), 0.81 (d, *J* = 6.4 Hz, 3H), 0.65 (d, *J* = 6.7 Hz, 3H); HRESIMS *m/z* 627.3398 [M − H]^−^ (calcd for C_33_H_49_N_4_O_8_, 627.3399).

### 3.8. Chemical Transformation of **4**

MeI (100 μL) was mixed to 1 mL of THF solution of compound **4** (2.0 mg). Then, NaH (0.5 mg) was added, and the mixture was stirred at room temperature for 2 h. After that, the reaction was quenched with aqueous HCl. The mixture was treated with EtOAc for three times. The EtOAc part was dried under vacuum pressure and subjected to HPLC having an ODS column with 60% acetonitrile/H_2_O to afford compound **4A** (2.8 mg, *t*_R_ = 13 min).

## 4. Conclusions

In summary, chemical investigation of the Antarctica sponge-derived fungus *Aspergillus insulicola* HDN151418 led to the isolation of three new aspochracin-type cyclic tripeptides, sclerotiotides M–O (**1**–**3**), along with the biogenetically related analogues, sclerotiotide L (**4**), sclerotiotide F (**5**), and sclerotiotide B (**6**). Among which, sclerotiotides M (**1**) and sclerotiotides N (**2)** represent the first example of aspochracin-type cyclic tripeptide, which was substituted by hexa-2,4-dienedioic acid/methyl ester moieties. Chemical derivatization indicated that compounds **4** and **5** could form from **6** during the fermentation or isolation steps. The antimicrobial activities of all the isolates were evaluated, and its structure–activity relationship (SAR) was also preliminary discussed. Our research results further expanded the members of the aspochracin-type cyclic tripeptide family, which again demonstrated that sponge-derived fungi are important producers of structurally diverse bioactive compounds.

## Figures and Tables

**Table 1:** ^1^H NMR (*δ*_H_, *J* in Hz) spectroscopic data for compounds **1**–**3** in DMSO-*d*_6_.

No.	1 *^a^*	2 *^b^*	3 *^b^*
2	4.50, q (7.1)	4.52, q (7.1)	4.65, q (6.4)
3	1.37, d (7.1)	1.39, d (7.1)	1.20, d (6.4)
N_Ala_-CH_3_	2.83, s	2.85, s	2.63, s
5	4.97, d (10.4)	4.99, d (10.3)	4.94, d (10.6)
6	2.21, m	2.22, m	2.28, m
7	0.63, d (6.7)	0.64, d (6.7)	0.74, d (6.7)
8	0.79, d (6.4)	0.80, d (6.3)	0.81, d (6.2)
N_Val_-CH_3_	2.83, s	2.85, s	2.91, s
10	4.71, m	4.73, m	5.33, t (5.5)
N_Orn (*α*)_H/ CH_3_	8.39, d (7.6)	8.46, d (7.5)	3.08, s
11	1.61. m	1.71, m	1.70, m
	1.96, m	1.96, m	1.95, m
12	1.46, m	1.47, m	1.48, m
	1.67, m	1.63, m	1.66, m
13	2.86, m	2.89, m	2.97, m
	3.01, m	3.01, m	3.12, m
N_Orn (*ω*)_H/CH_3_	7.47, t (6.0)	7.51, t (5.7)	2.83, s
2′	6.55, d (14.3)	6.60, d (14.6)	6.64, d (15.0)
3′	7.12, ov.	7.17, ov.	7.11, dd (15.0, 11.2)
4′	7.16, ov.	7.26, ov.	6.46, dd (15.5, 11.2)
5′	6.22, d (14.4)	6.36, d (14.8)	6.01, dd (15.5, 7.1)
6′			3.71, dd (7.1, 3.9)
7′		3.69, s	3.34, m
8′			1.02, d (6.4)
9′			3.23, s
10′			3.25, s
COOH	12.5, brs		

*^a^* Recorded at 500 MHz. *^b^* Recorded at 400 MHz. *ov.* Overlapped signal.

**Table 2:** ^13^C NMR spectroscopic data for compounds **1**–**3** in DMSO-*d*_6_.

No.	1 *^a^*	2 *^b^*	3 *^c^*
1	171.1	171.2	168.5
2	54.9	55.0	52.9
3	16.7	16.7	17.2
N_Ala_-CH_3_	30.1	30.1	28.7
4	169.5	169.6	168.2
5	58.0	58.1	57.6
6	26.8	26.9	26.3
7	18.1	18.2	18.1
8	20.2	20.3	19.9
N_Val_-CH_3_	30.2	30.3	29.7
9	171.9	172.0	171.8
10	50.1	50.2	52.7
N_Orn (*α*)_-CH_3_			31.7
11	28.4	28.4	23.0
12	23.1	23.2	24.5
13	39.5	39.5	47.3
N_Orn (*ω*)_-CH_3_			33.6
1′	163.8	163.8	166.6
2′	132.3	133.0	122.3
3′	141.8	142.5	141.7
4′	136.7	136.7	131.5
5′	128.2	126.8	138.8
6′	167.5	166.7	83.9
7′		52.1	78.9
8′			15.6
9′			57.1
10′			56.8

*^a^* Recorded at 125 MHz. *^b^* Recorded at 150 MHz. *^c^* Recorded at 100 MHz.

**Table 3:** Antimicrobial assays of compounds **1**–**5** (MIC μM).

No.	*B. cereus*	*P. species*	*M. phlei*	*E. tarda*	*B. subtilis*	MRCNS	MRSA	*V. parahemolyticus*
**1**	3.13	3.13	3.13	1.56	6.25	12.5	25.0	3.13
**2**	6.25	6.25	12.5	1.56	12.5	25.0	25.0	6.25
**3**	>50.0	>50.0	>50.0	25.0	>50.0	>50.0	>50.0	25.0
**4**	25.0	25.0	>50.0	25.0	>50.0	>50.0	>50.0	25.0
**5**	25.0	25.0	>50.0	25.0	>50.0	>50.0	>50.0	25.0
**6**	>50.0	>50.0	>50.0	>50.0	>50.0	>50.0	>50.0	>50.0
CIP *^a^*	0.780	0.195	0.780	0.0125	0.195	25.0	25.0	0.390

*^a^* Ciprofloxacin was used as positive drug.

## Supplementary Materials

The following are available online at https://www.mdpi.com/1660-3397/18/11/532/s1: Figure S1: Structures of aspochracin-type cyclic tripeptides, Figure S2: HPLC analysis of the crude of *Aspergillus insulicola* HDN151418; Figure S3: The 18S rRNA sequences data of *Aspergillus insulicola* HDN151418; Figures S4–S38: 1D and 2D NMR spectra, HRESIMS spectra, IR spectra of compounds **1**–**4**; Table S1. ^1^H NMR (400 MHz) spectroscopic data for compound **4**. Figures S39–S40: ^1^H NMR spectra of *R*- and *S*- MPA esters of **4**. Figure S41. ECD spectra of **3**, **4**, **4A**. Figure S42. HSQMBC spectrum of **3**. Table S1: ^1^H NMR parameters of **1**–**4**. Table S2: ^13^C NMR parameters of **1**–**4**.

## References

[1] Carroll A.R., Copp B.R., Davis R.A., Keyzers R.A., Prinsep M.R. (2020). Marine natural products. Nat. Prod. Rep..

[2] Carroll A.R., Copp B.R., Davis R.A., Keyzers R.A., Prinsep M.R. (2019). Marine natural products. Nat. Prod. Rep..

[3] Skropeta D., Wei L. (2014). Recent advances in deep-sea natural products. Nat. Prod. Rep..

[4] Rateb M.E., Ebel R. (2011). Secondary metabolites of fungi from marine habitats. Nat. Prod. Rep..

[5] Velle A., Cebollada A., Macias R., Iglesias M., Gil-Moles M., Sanz Miguel P.J. (2017). From imidazole toward imidazolium salts and *N*-heterocyclic carbene ligands: Electronic and geometrical redistribution. ACS Omega.

[6] Wu Y., Liao H., Liu L.Y., Sun F., Chen H.F., Jiao W.H., Zhu H.R., Yang F., Huang G., Zeng D.Q. (2020). Phakefustatins A-C: Kynurenine-bearing cycloheptapeptides as RXRalpha modulators from the marine sponge *Phakellia Fusca*. Org. Lett..

[7] Xu W.J., Liao X.J., Xu S.H., Diao J.Z., Pan S.S. (2010). Isolation, structure determination, and synthesis of galaxamide, a rare cytotoxic cyclic pentapeptide from a marine algae *Galaxaura filamentosa*. Org. Lett..

[8] Teta R., Marteinsson V.T., Longeon A., Klonowski A.M., Groben R., Bourguet-Kondracki M.L., Costantino V., Mangoni A. (2017). Thermoactinoamide A, an antibiotic lipophilic cyclopeptide from the icelandic thermophilic bacterium *Thermoactinomyces vulgaris*. J. Nat. Prod..

[9] Skehan P., Storeng R., Scudiero D., Monks A., McMahon J., Vistica D., Warren J.T., Bokesch H., Kenney S., Boyd M.R. (1990). New colorimetric cytotoxicity assay for anticancer-drug screening. J. Natl. Cancer Inst..

[10] Zheng J., Xu Z., Wang Y., Hong K., Liu P., Zhu W. (2010). Cyclic tripeptides from the halotolerant fungus *Aspergillus sclerotiorum* PT06-1. J. Nat. Prod..

[11] Motohashi K., Inaba S., Takagi M., Shin-ya K. (2009). JBIR-15, a new aspochracin derivative, isolated from a sponge-derived fungus, *Aspergillus sclerotiorum Huber* Sp080903f04. Biosci. Biotechnol. Biochem..

[12] Liu J., Gu B., Yang L., Yang F., Lin H. (2018). New Anti-inflammatory cyclopeptides from a sponge-derived fungus *Aspergillus violaceofuscus*. Front. Chem..

[13] Myokei R., Sakurai A., Chang C.F., Kodaira Y., Tamura S. (1969). Structure of aspochracin, an insecticidal metabolite of Aspergillus ochraceus. Tetrahedron Lett..

[14] Zhou H., Li L., Wu C., Kurtan T., Mandi A., Liu Y., Gu Q., Zhu T., Guo P., Li D. (2016). Penipyridones A-F, pyridone alkaloids from *Penicillium funiculosum*. J. Nat. Prod..

[15] Zhou H., Li L., Wang W., Che Q., Li D., Gu Q., Zhu T. (2015). Chrodrimanins I and J from the antarctic moss-derived fungus *Penicillium funiculosum* GWT2-24. J. Nat. Prod..

[16] Shah M., Sun C., Sun Z., Zhang G., Che Q., Gu Q., Zhu T., Li D. (2020). Antibacterial polyketides from antarctica sponge-derived fungus *Penicillium* sp. HDN151272. Mar. Drugs.

[17] Wu G., Lin A., Gu Q., Zhu T., Li D. (2013). Four new chloro-eremophilane sesquiterpenes from an Antarctic deep-sea derived fungus, *Penicillium* sp. PR19N-1. Mar. Drugs.

[18] Marfey P. (1984). Determination of D-amino acids. II. Use of a bifunctional reagent, 1,5-difluoro-2,4-dinitrobenzene. Carlsberg Res. Commun..

[19] Sun C., Ge X., Mudassir S., Zhou L., Yu G., Che Q., Zhang G., Peng J., Gu Q., Zhu T. (2019). New glutamine-containing azaphilone alkaloids from deep-sea-derived fungus *Chaetomium globosum* HDN151398. Mar. Drugs.

[20] Latypov S.K., Seco J.M., Quinoa E., Riguera R. (1996). MTPA vs MPA in the determination of the absolute configuration of chiral alcohols by ^1^H NMR. J. Org. Chem..

[21] Mosmann T. (1983). Rapid colorimetric assay for cellular growth and survival: Application to proliferation and cytotoxicity assays. J. Immunol. Methods.

[22] Andrews J.M. (2001). Determination of minimum inhibitory concentrations. J. Antimicrob. Chemother..

[23] Yu G., Sun Z., Peng J., Zhu M., Che Q., Zhang G., Zhu T., Gu Q., Li D. (2019). Secondary metabolites produced by combined culture of *Penicillium crustosum* and a *Xylaria* sp.. J. Nat. Prod..

