# Supplementary material for: Antibacterial Cyclic Tripeptides from Antarctica-Sponge-Derived Fungus Aspergillus insulicola HDN151418
Source: Mar Drugs. 2020 Oct 26;18(11):532. doi: 10.3390/md18110532 (PMC7694092; doi:10.3390/md18110532)
Supplement: Supplementary file 1 [file marinedrugs-18-00532-s001.pdf]

Supporting Information for

Antibacterial Cyclic Tripeptides from Antarctica-Sponge-Derived Fungus *Aspergillus insulicola* HDN151418

Chunxiao Sun [1], Ziping Zhang [1], Zilin Ren [1], Liu Yu [1], Huan Zhou [1], Yaxin Han [1], Mudassir Shah [1], Qian Che [1], Guojian Zhang [1,2], Dehai Li [1,2,3,*], and Tianjiao Zhu [1,*]

[1] Key Laboratory of Marine Drugs, Chinese Ministry of Education, School of Medicine and Pharmacy, Ocean University of China, Qingdao 266003, P. R. China

[2] Laboratory for Marine Drugs and Bioproducts of Qingdao National Laboratory for Marine Science and Technology, Qingdao, 266237, P. R. China

[3] Open Studio for Druggability Research of Marine Natural Products, Pilot National Laboratory for Marine Science and Technology, Qingdao, 266237, P. R. China

***** Correspondence: dehaili@ouc.edu.cn (D.L.); Tel.: 0086-532-82031619; zhutj@ouc.edu.cn (T.Z.); Tel.: 0086-532-82031632

Table of Contents

Figure S1. Structures of aspochracin-type cyclic tripeptides. 3
Figure S2. HPLC analysis of the crude of *Aspergillus insulicola* HDN151418....................... 3
Figure S3. The 18S rRNA sequences data of *Aspergillus insulicola* HDN151418.................. 4
Figure S4. ^{1}H NMR (500 MHz, DMSO-d_6) spectrum of compound **1**..................................... 4
Figure S5. ^{13}C NMR (125 MHz, DMSO-d_6) spectrum of compound **1**.................................. 5
Figure S6. DEPT (125 MHz, DMSO-d_6) spectrum of compound **1**.. 5
Figure S7. ^{1}H-^{1}H COSY spectrum of compound **1**. 6
Figure S8. HSQC spectrum of compound **1**.. 6
Figure S9. ^{1}H-^{13}C HMBC spectrum of compound **1**. 7
Figure S10. NOESY spectrum of compound **1**. 7
Figure S11. HRESIMS spectrum of compound **1**. 8
Figure S12. IR spectrum of compound **1**.. 8
Figure S13. ^{1}H NMR (400 MHz, DMSO-d_6) spectrum of compound **2**.................................. 9
Figure S14. ^{13}C NMR (150 MHz, DMSO-d_6) spectrum of compound **2**................................. 9
Figure S15. DEPT (150 MHz, DMSO-d_6) spectrum of compound **2**.................................... 10
Figure S16. ^{1}H-^{1}H COSY spectrum of compound **2**. 10
Figure S17. HSQC spectrum of compound **2**... 11
Figure S18. ^{1}H-^{13}C HMBC spectrum of compound **2**. 11
Figure S19. NOESY spectrum of compound **2**. 12
Figure S20. HRESIMS spectrum of compound **2**. 12
Figure S21. IR spectrum of compound **2**.. 13
Figure S22. ^{1}H NMR (400 MHz, DMSO-d_6) spectrum of compound **3**................................ 13
Figure S23. ^{13}C NMR (100 MHz, DMSO-d_6) spectrum of compound **3**............................... 14
Figure S24. ^{1}H-^{1}H COSY spectrum of compound **3**. 14
Figure S25. HSQC spectrum of compound **3**... 15
Figure S26. ^{1}H-^{13}C HMBC spectrum of compound **3**. 15
Figure S27. NOESY spectrum of compound **3**. 16
Figure S28. HRESIMS spectrum of compound **3**. 16
Figure S29. IR spectrum of compound **3**.. 17
Table S1. ^{1}H NMR (400 MHz) spectroscopic data for compound **4**. 17
Figure S30. ^{1}H NMR (400 MHz, $CDCl_3$) spectrum of compound **4**. 18
Figure S31. ^{1}H NMR (400 MHz, DMSO-d_6) spectrum of compound **4**................................ 18
Figure S32. ^{13}C NMR (150 MHz, DMSO-d_6) spectrum of compound **4**............................... 19
Figure S33. ^{1}H-^{1}H COSY spectrum of compound **4**. 19
Figure S34. HSQC spectrum of compound **4**... 20
Figure S35. ^{1}H-^{13}C HMBC spectrum of compound **4**. 20
Figure S36. NOESY spectrum of compound **4**. 21
Figure S37. HRESIMS spectrum of compound **4**. 21
Figure S38. IR spectrum of compound **4**.. 22
Figure S39. ^{1}H NMR (400 MHz, DMSO-d_6) spectrum of *R*-MPA ester of **4** (**4g**)................. 22
Figure S40. ^{1}H NMR (400 MHz, DMSO-d_6) spectrum of *S*-MPA ester of **4** (**4h**)................. 23
Figure S41. ECD spectra of **3**, **4A**. 23
Figure S42. HSQMBC spectrum of **3**.. 24

Table S2. ^{1}H NMR parameters of **1-4**. 24
Table S3. ^{13}C NMR parameters of **1-4**. 25

Figure S1. Structures of aspochracin-type cyclic tripeptides.

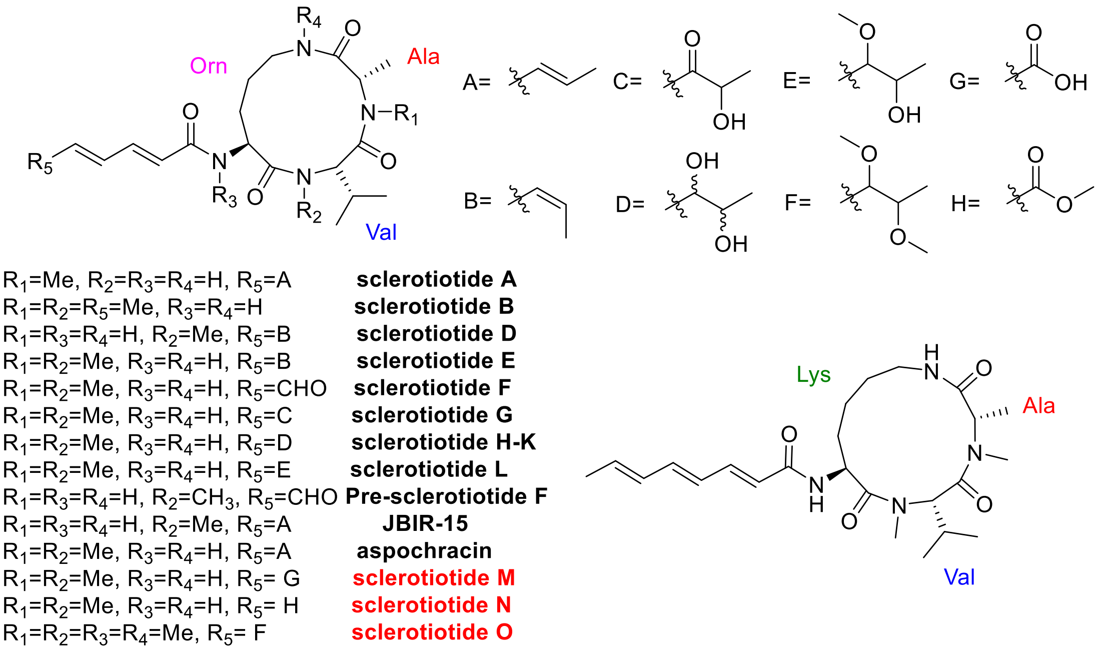

Figure S2. HPLC analysis of the crude of *Aspergillus insulicola* HDN151418.

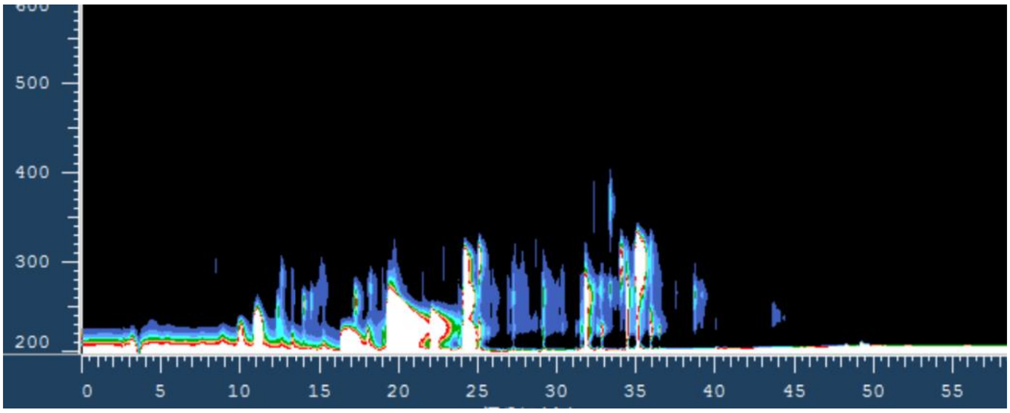

Figure S3. The 18S rRNA sequences data of *Aspergillus insulicola* HDN151418.

GGGGCCATCCTCCCACCCTTGTATACCGTACCAAGTTGCTTCGGCGGGCCC
GCCGTTCGCGCGGCCGCCGGGGGGGAACCCCTCCCCCCGGGCGAGCGCCC
GCCGGAGACCCCAACGTGAACACTGTCTGAAGTTTTGTCGTCTGAGTTCG
ATTGTATCGCAATCAGTTAAAACTTTCAACAATGGATCTCTTGGTTCCGGCA
TCGATGAAGAACGCAGCGAAATGCGATAATTAATGTGAATTGCAGAATTCA
GTGAATCATCGAGTCTTTGAACGCACATTGCACCCCCTGGTATTCCGGGGG
GTATGCCTGTCCGAGCGTCATTGCTGCCCTCAAGCCCGGCTTGTGTGTTGG
GTCCTCGTCCCCCCCCCCCCGGGGGGGACGGGCCCGAAAGGCAGCGGCG
GCACCGCGTCCGGTCCTCGAGCGTATGGGGCTTTGTCACACCGCTCTCGTA
GGCCCGGCCGGCGCTGGCCGACGCTGAAAAGCAACCATCATTTCTCCAGG
TTGACCTCGGATCAGGTAGGGATACCCGCTGAACTTAAG

Figure S4. ^{1}H NMR (500 MHz, DMSO-d_6) spectrum of compound **1**.

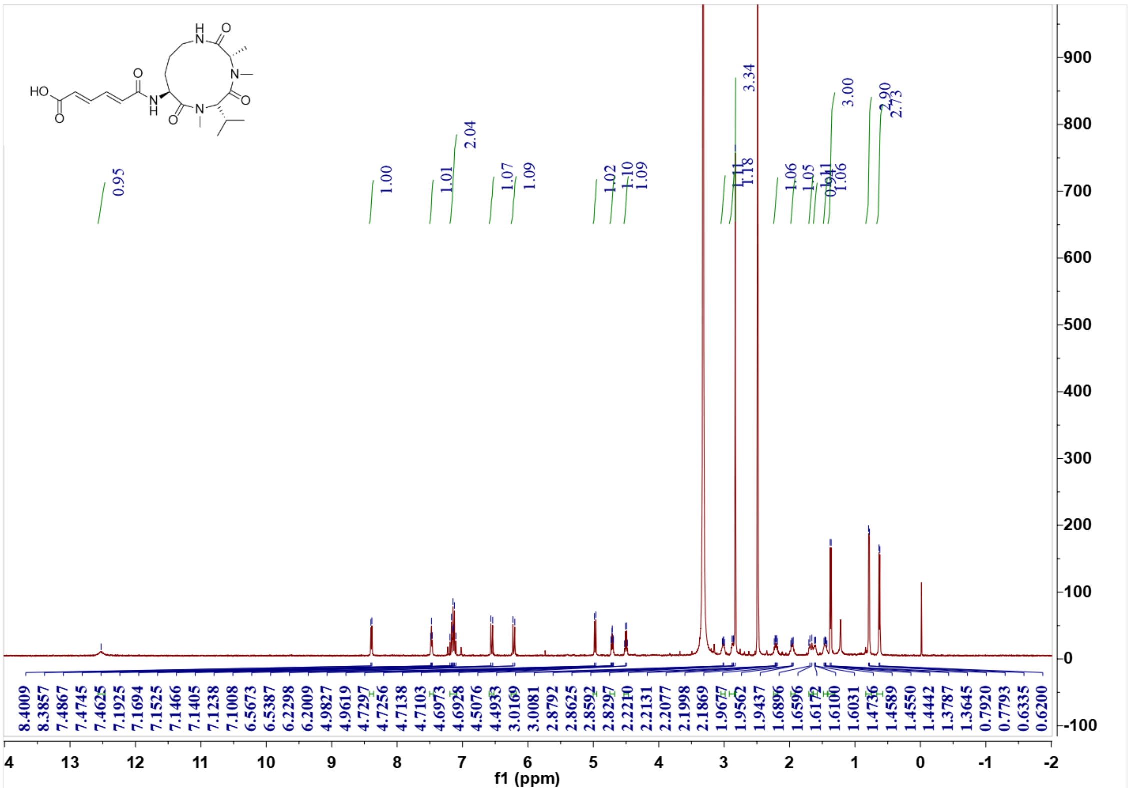

Figure S5. ^{13}C NMR (125 MHz, DMSO-d_6) spectrum of compound **1**.

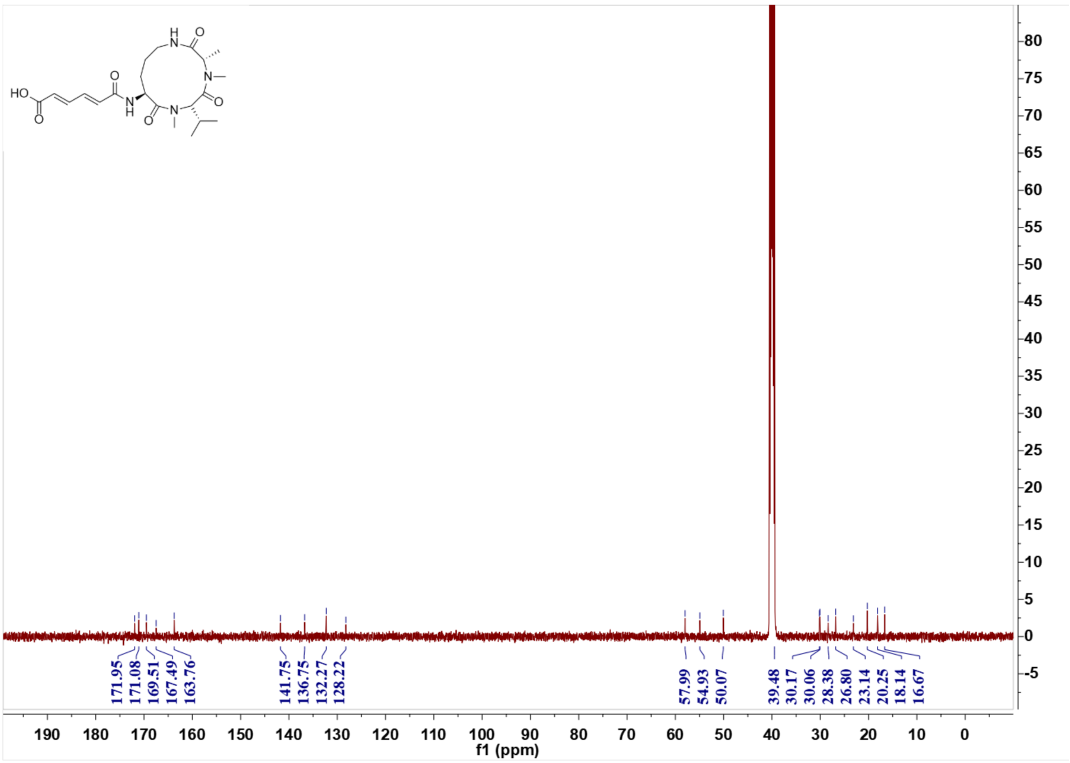

Figure S6. DEPT (125 MHz, DMSO-d_6) spectrum of compound **1**.

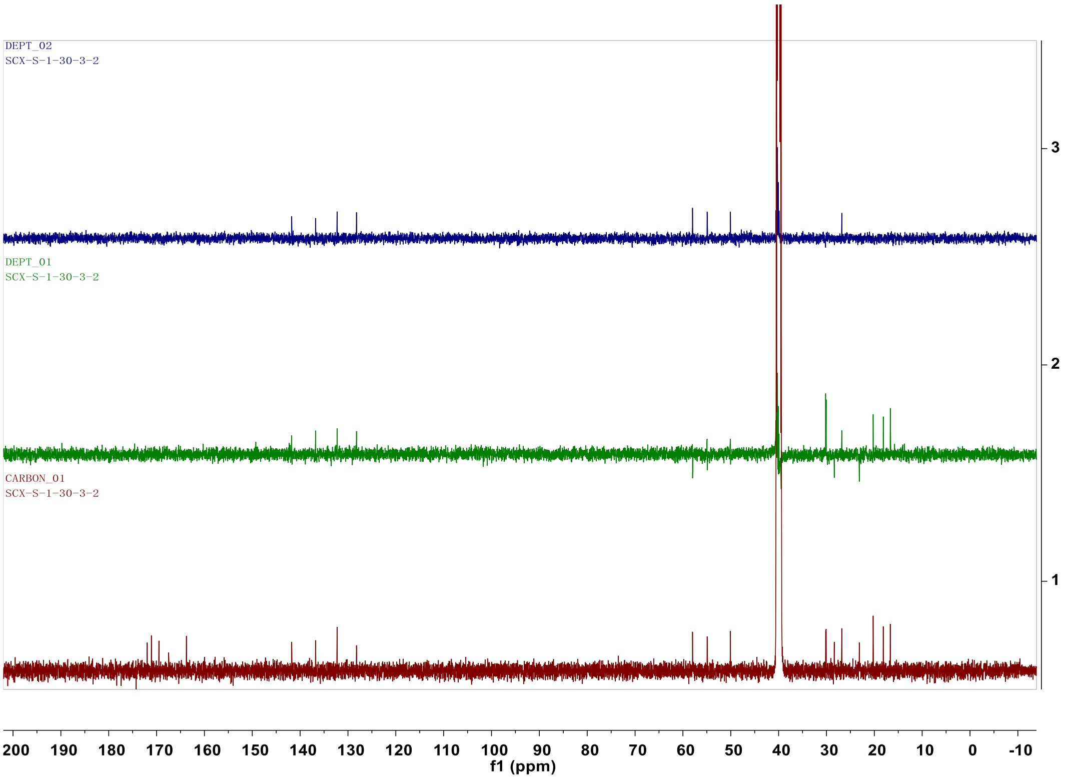

Figure S7. ^{1}H-^{1}H COSY (500 MHz, DMSO-d_6) spectrum of compound **1**.

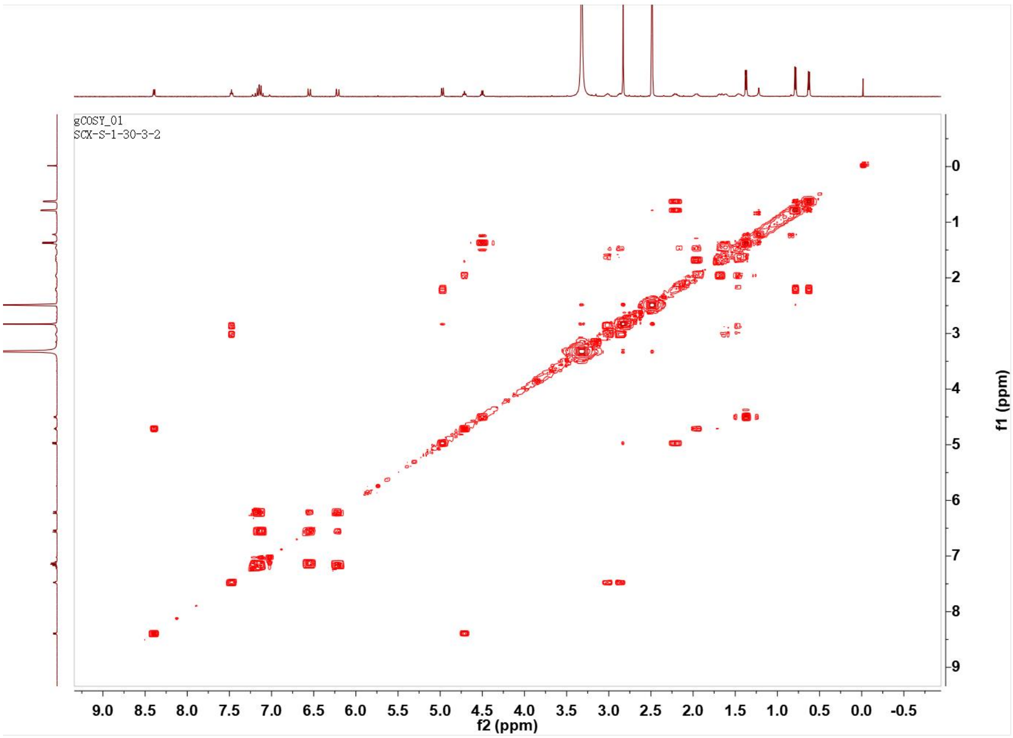

Figure S8. HSQC (500 MHz, DMSO-d_6) spectrum of compound **1**.

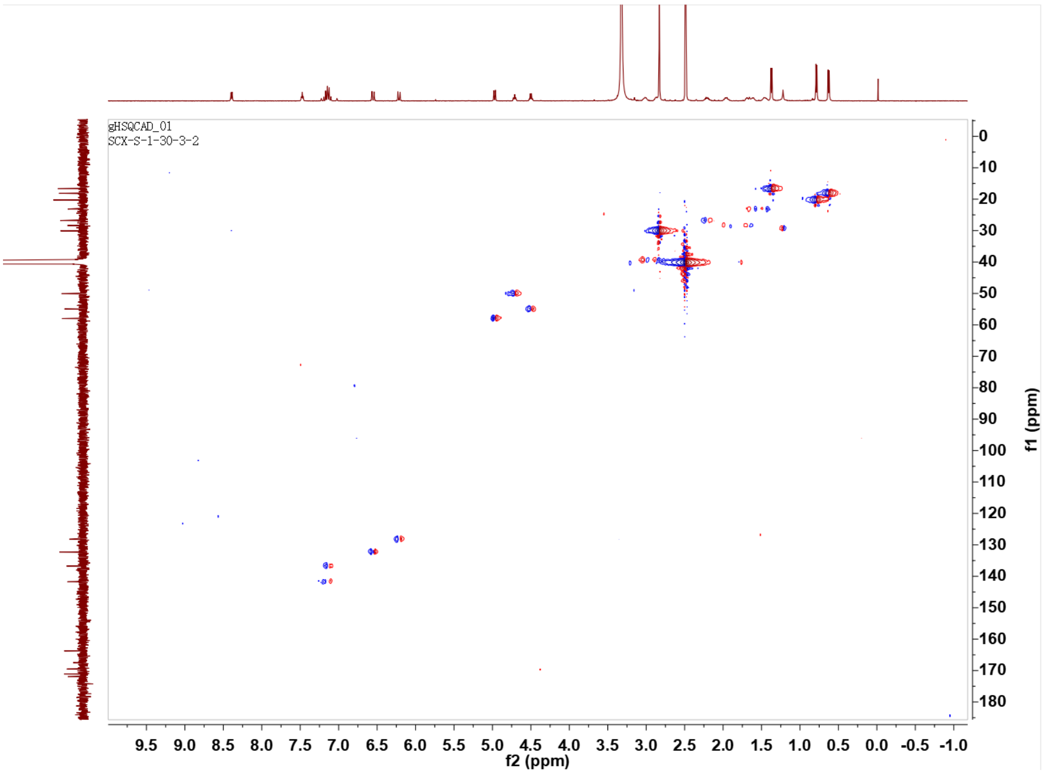

Figure S9. ^{1}H-^{13}C HMBC (500 MHz, DMSO-d_6) spectrum of compound **1**.

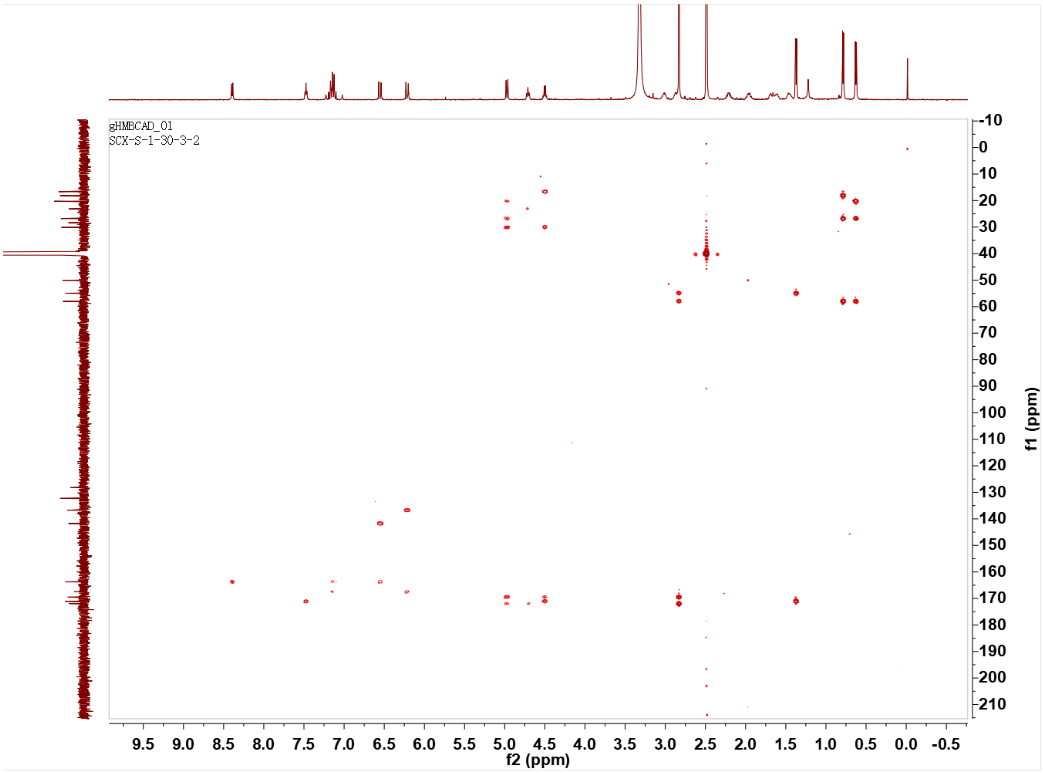

Figure S10. NOESY (500 MHz, DMSO-d_6) spectrum of compound **1**.

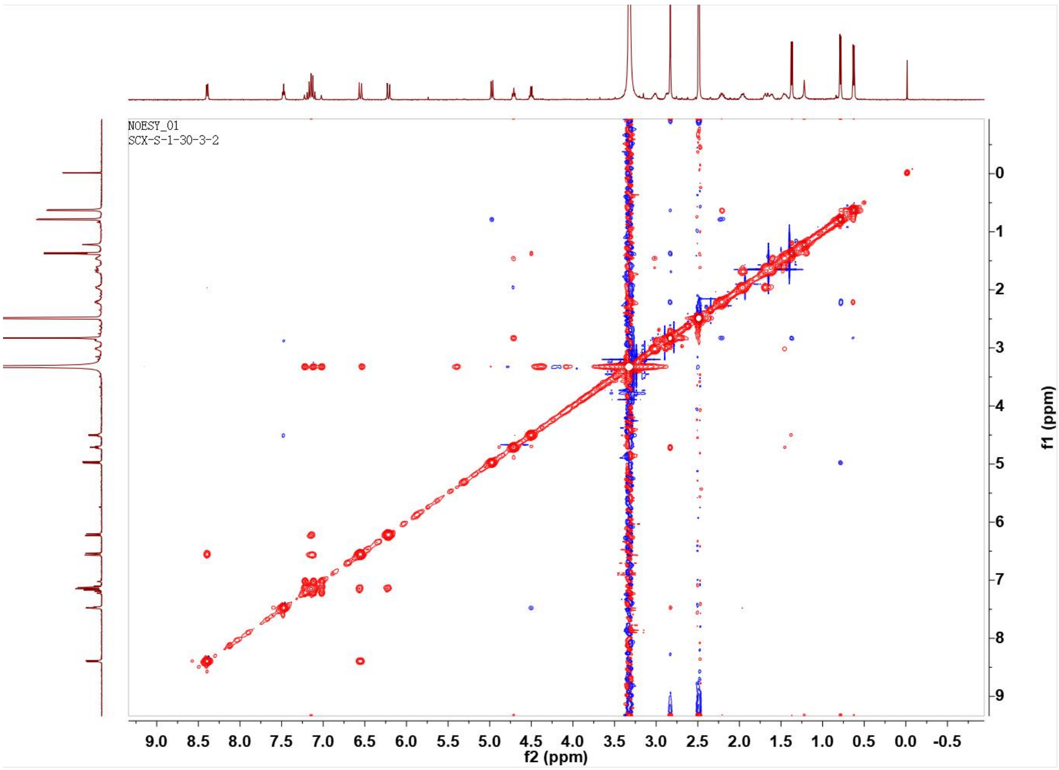

Figure S11. HRESIMS spectrum of compound **1**.

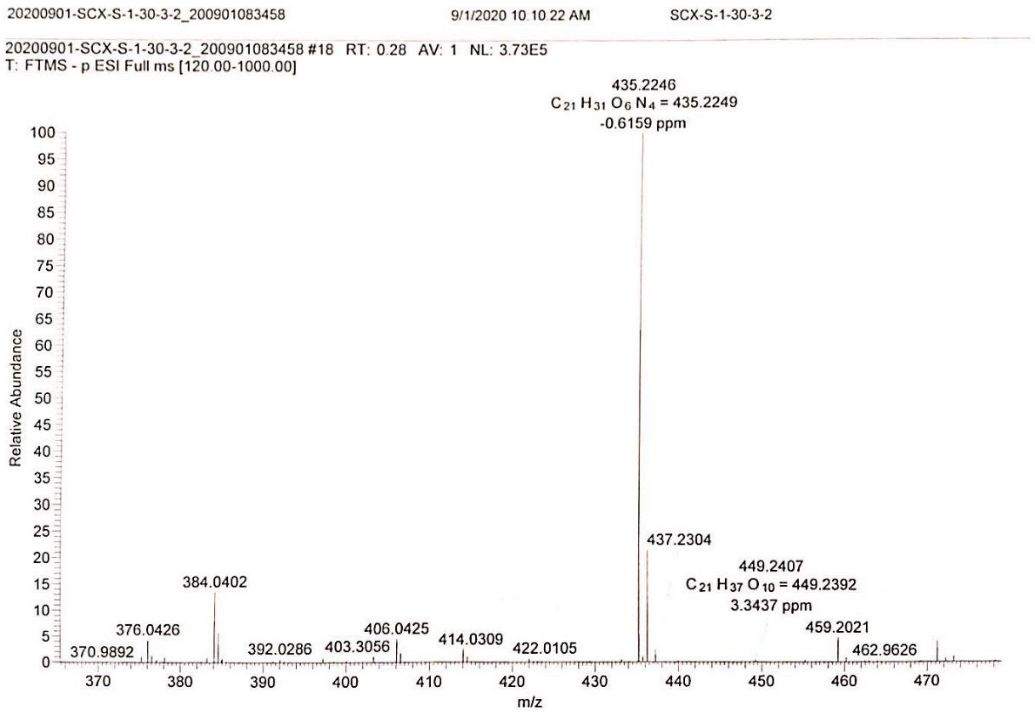

Figure S12. IR spectrum of compound **1**.

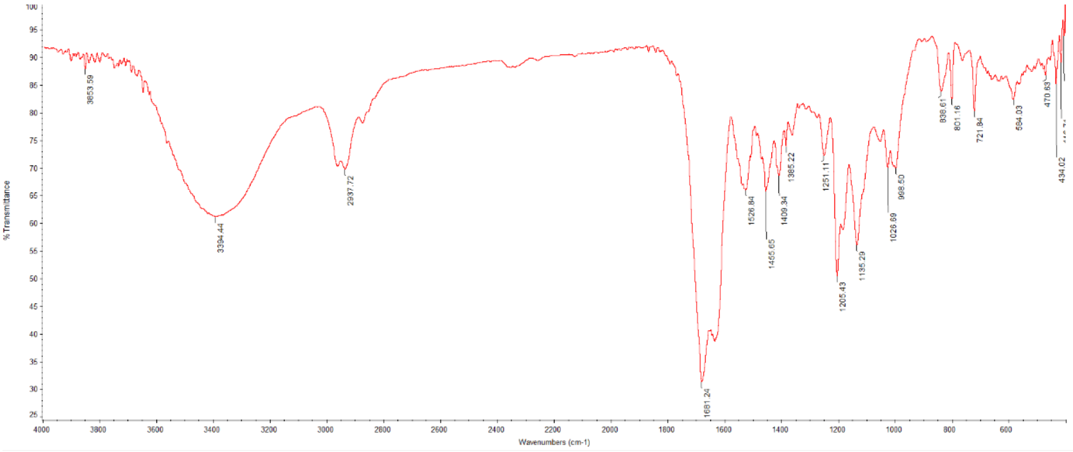

Figure S13. ^{1}H NMR (400 MHz, DMSO-d_6) spectrum of compound **2**.

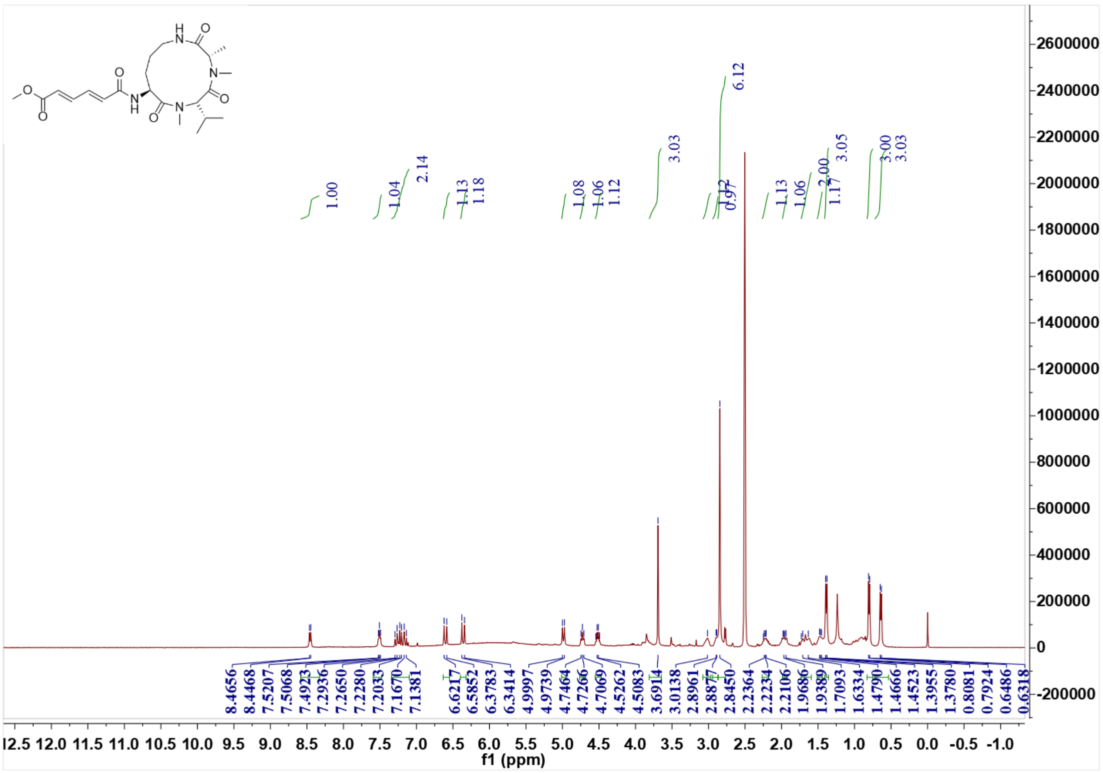

Figure S14. ^{13}C NMR (150 MHz, DMSO-d_6) spectrum of compound **2**.

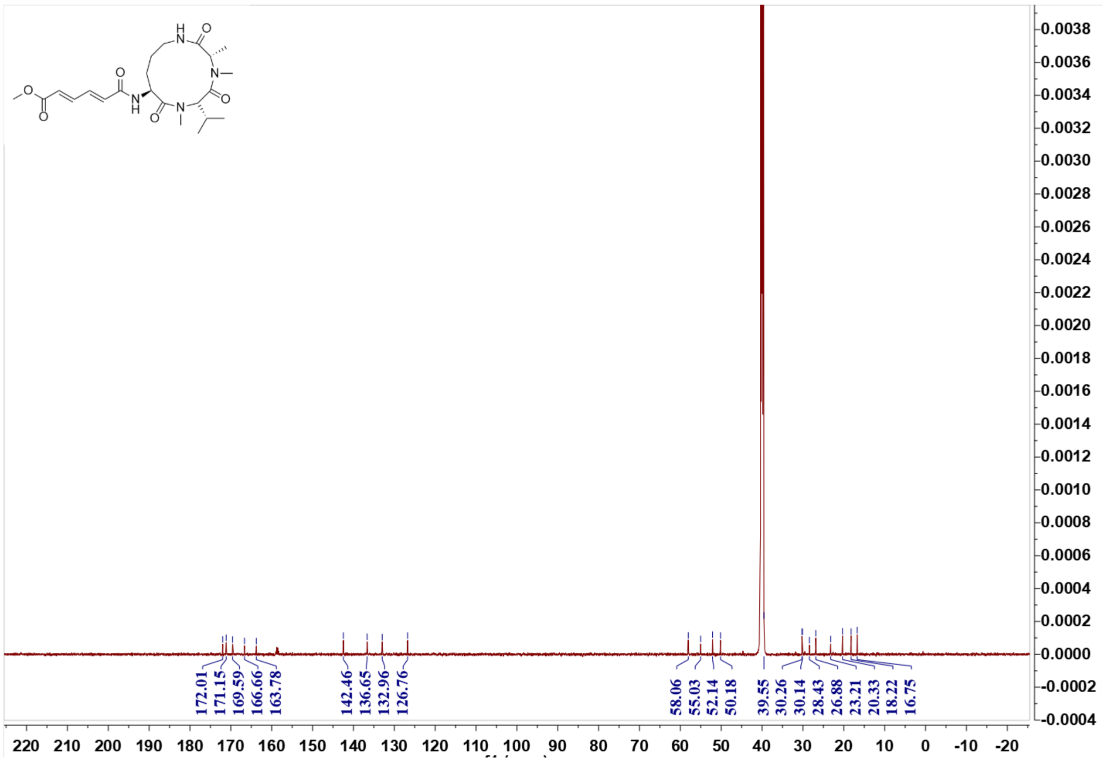

Figure S15. DEPT (150 MHz, DMSO-d_6) spectrum of compound **2**.

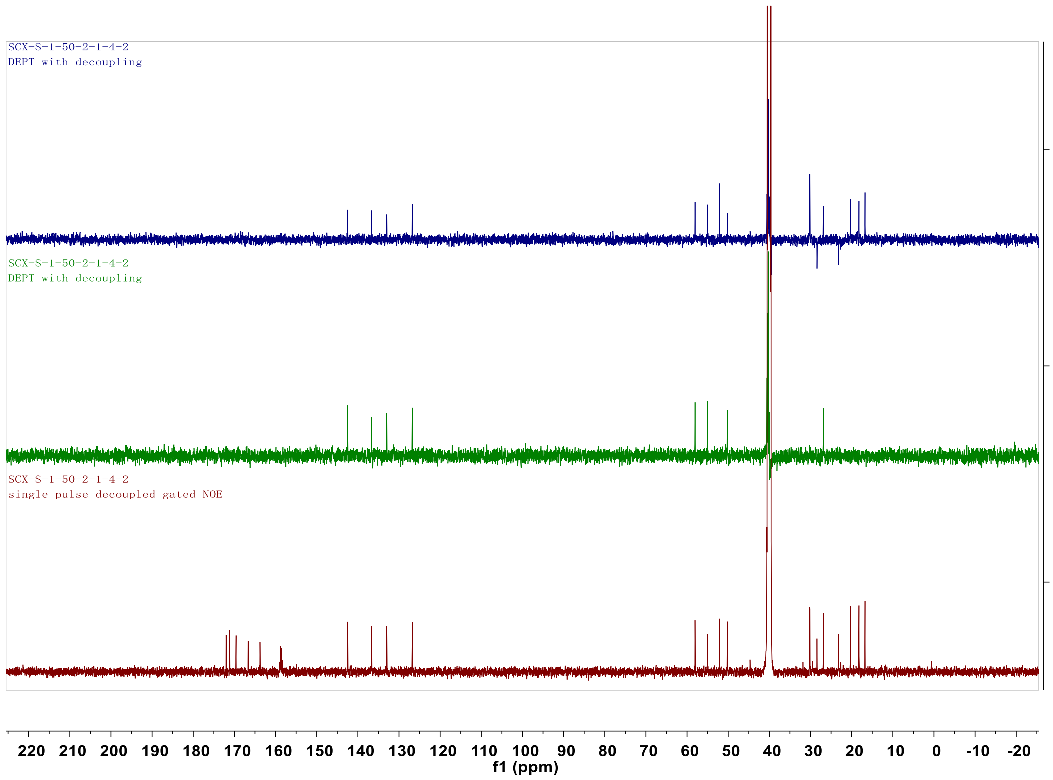

Figure S16. ^{1}H-^{1}H COSY (600 MHz, DMSO-d_6) spectrum of compound **2**.

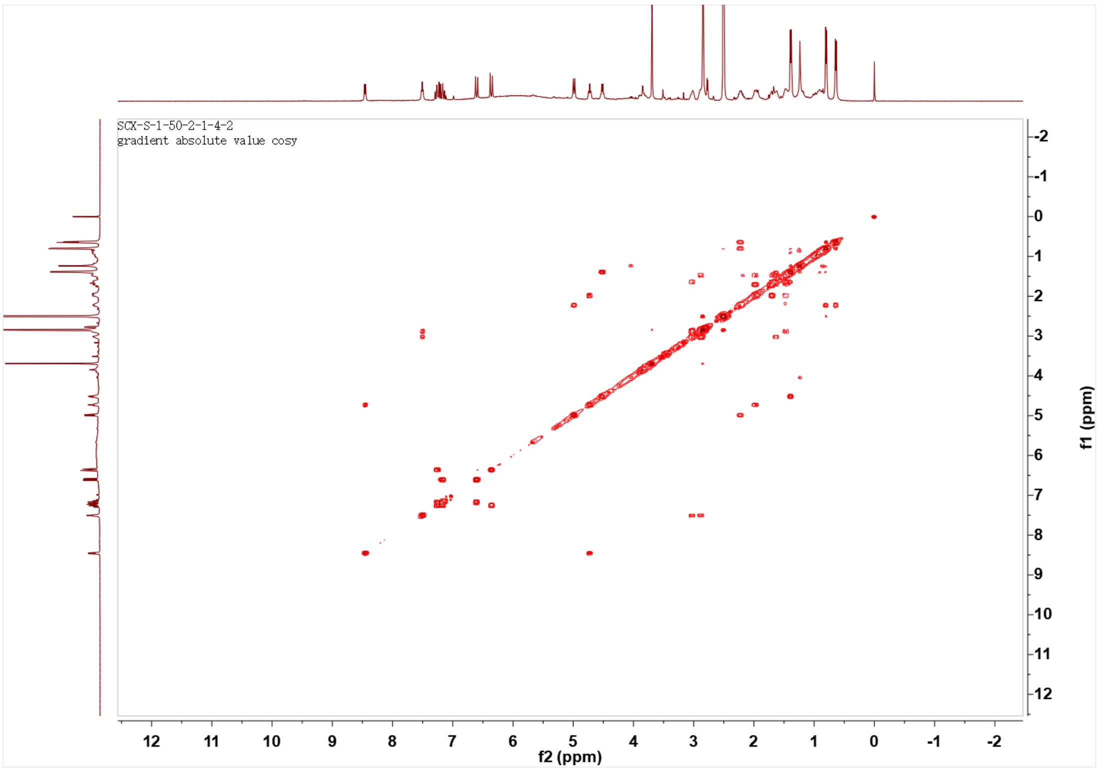

Figure S17. HSQC (600 MHz, DMSO-d_6) spectrum of compound **2**.

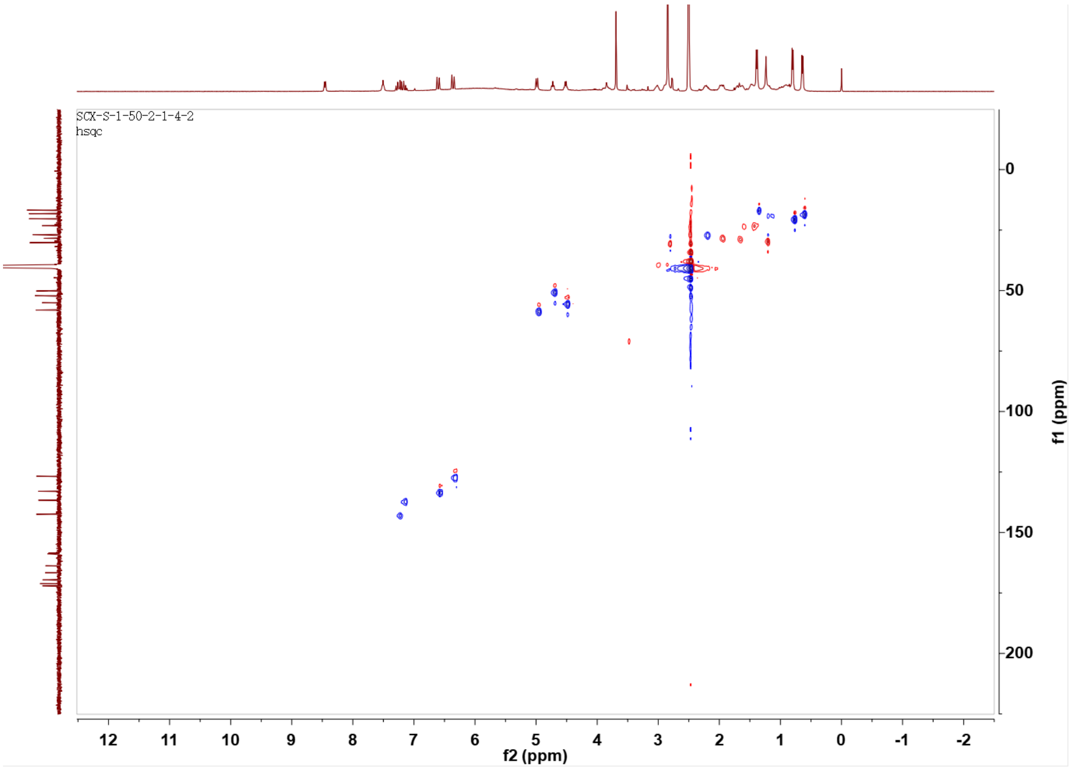

Figure S18. ^{1}H-^{13}C HMBC (600 MHz, DMSO-d_6) spectrum of compound **2**.

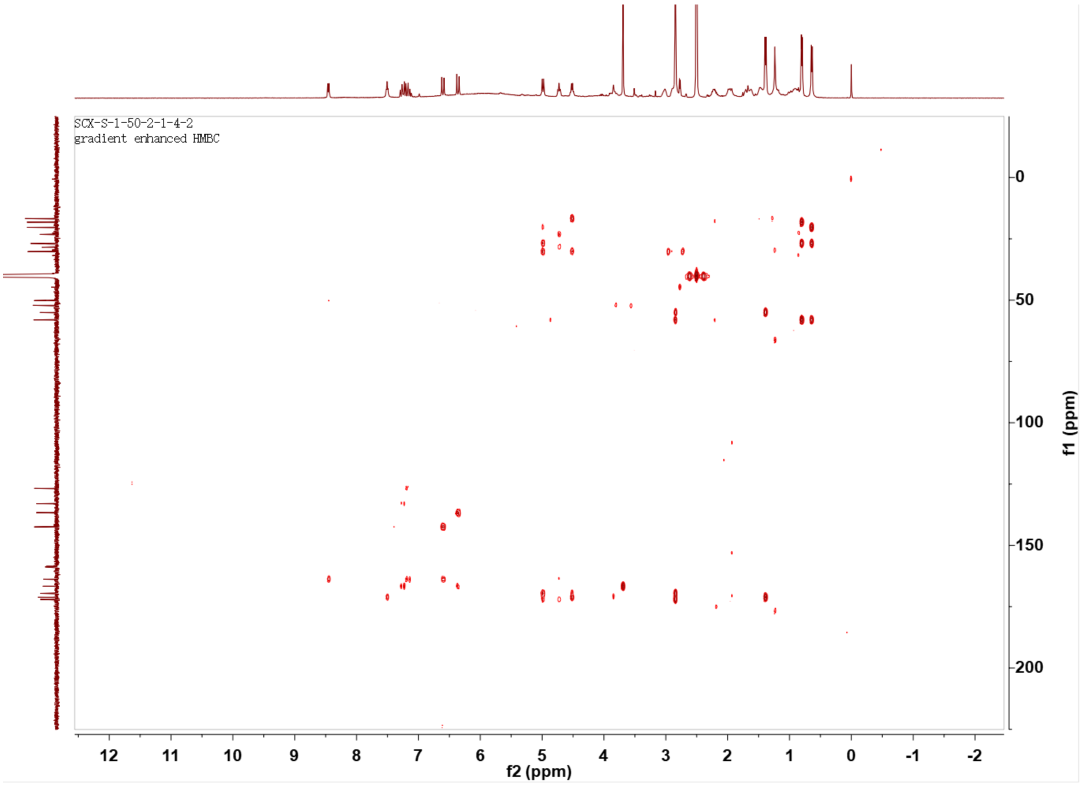

Figure S19. NOESY (500 MHz, DMSO-d_6) spectrum of compound **2**.

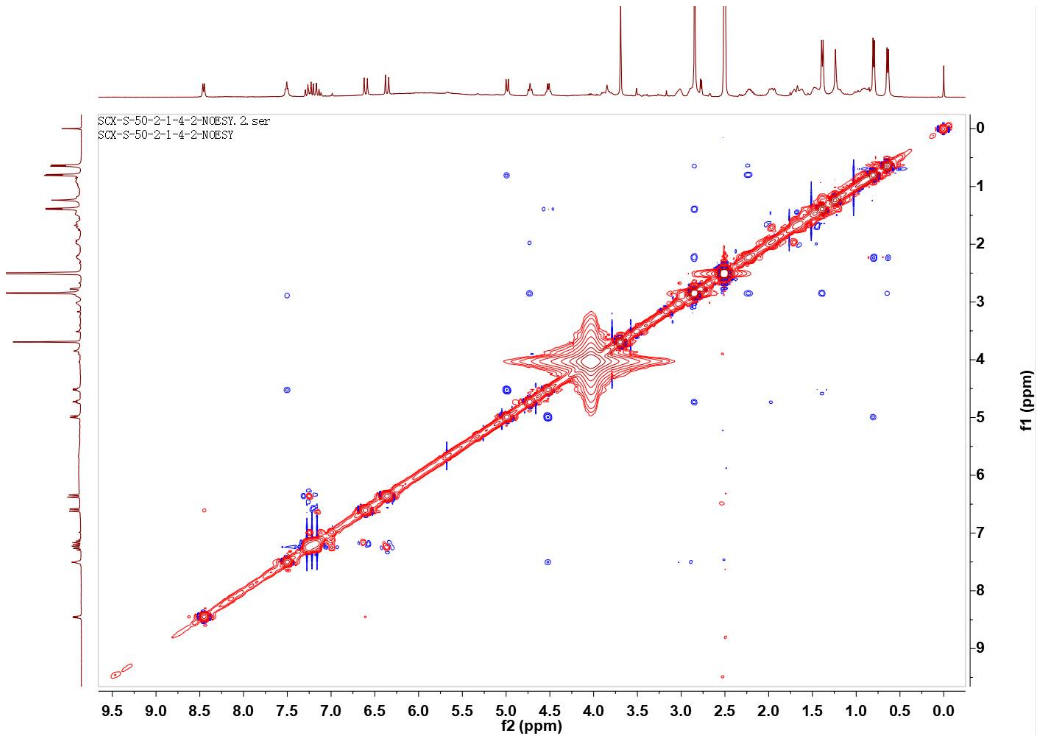

Figure S20. HRESIMS spectrum of compound **2**.

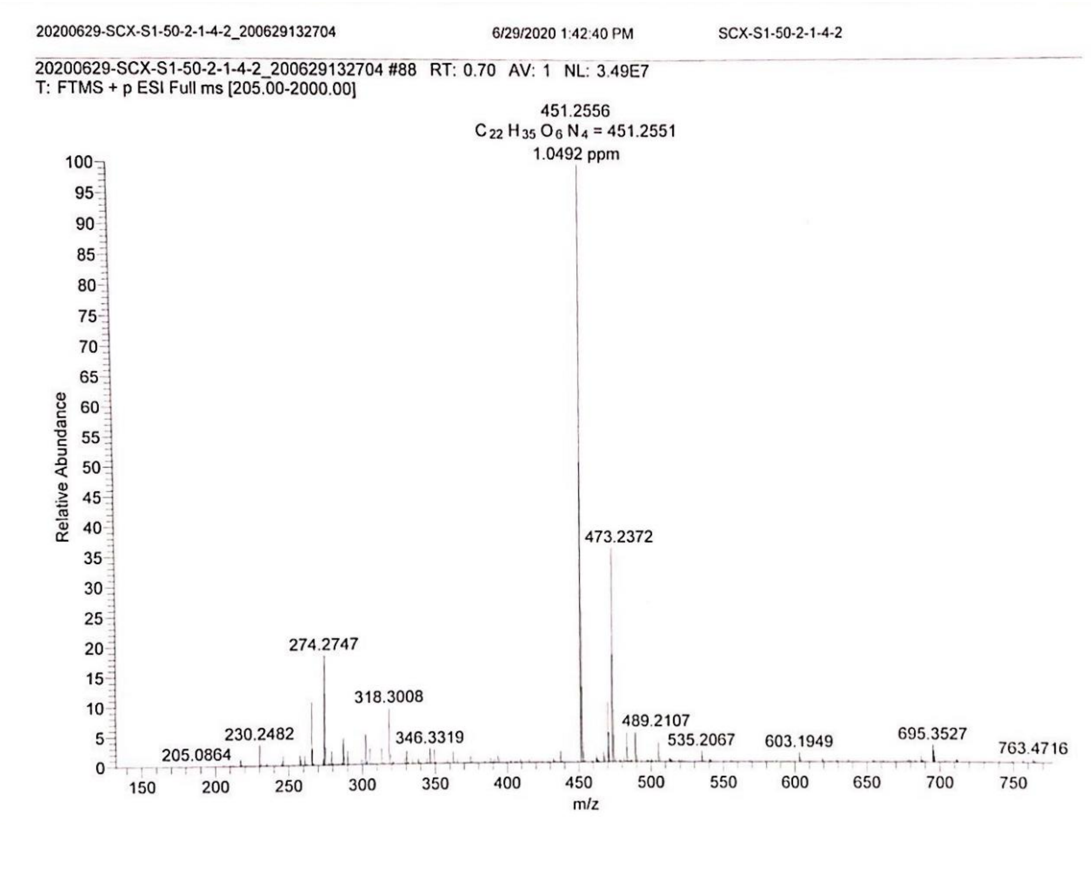

Figure S21. IR spectrum of compound **2**.

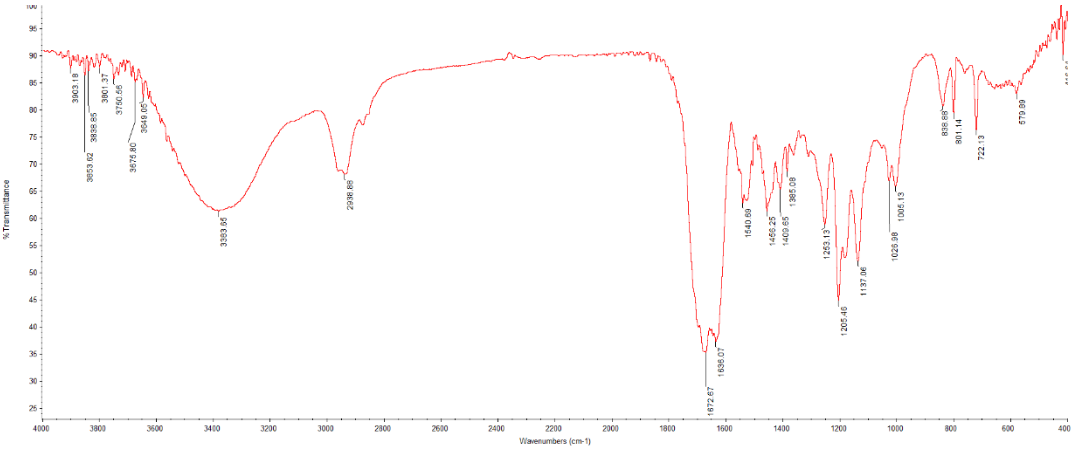

Figure S22. ^{1}H NMR (400 MHz, DMSO-d_6) spectrum of compound **3**.

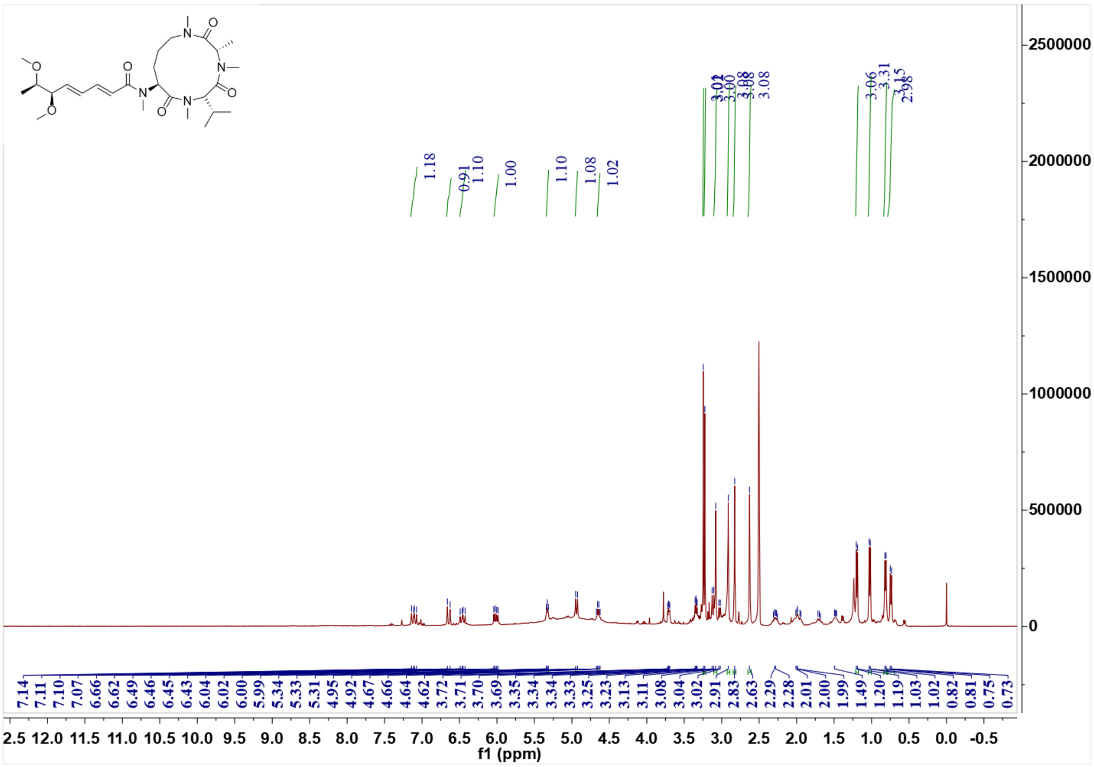

Figure S23. ^{13}C NMR (100 MHz, DMSO-d_6) spectrum of compound **3**.

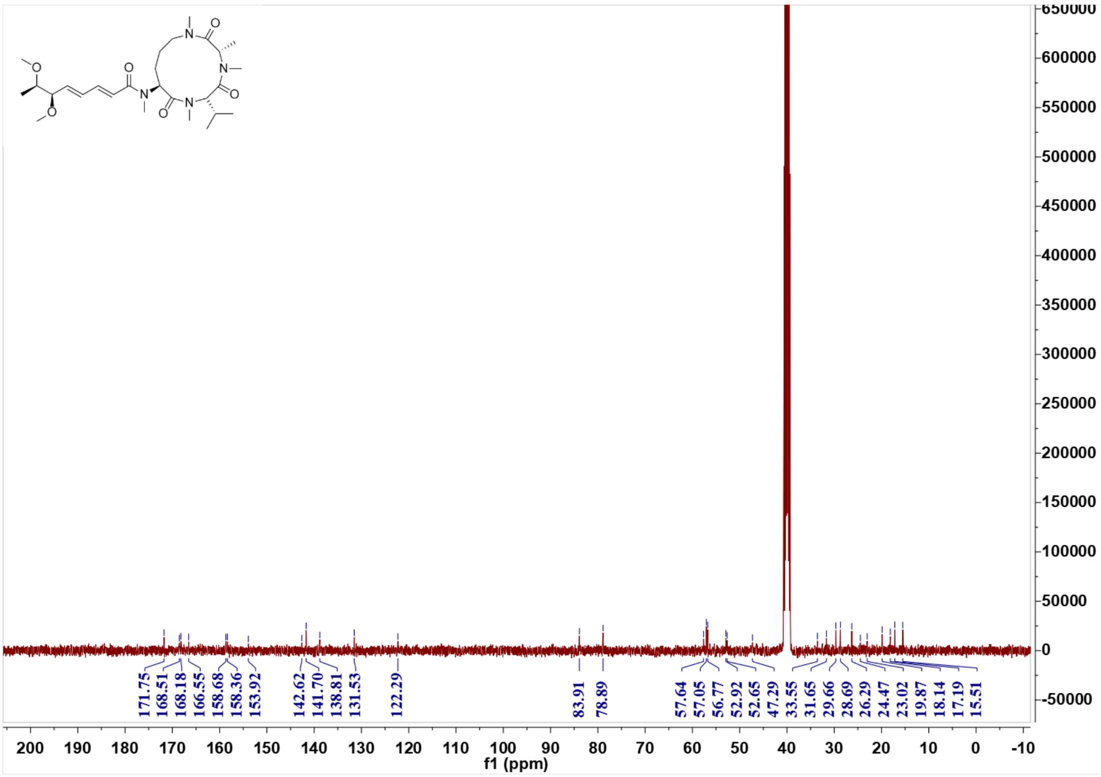

Figure S24. ^{1}H-^{1}H COSY (400 MHz, DMSO-d_6) spectrum of compound **3**.

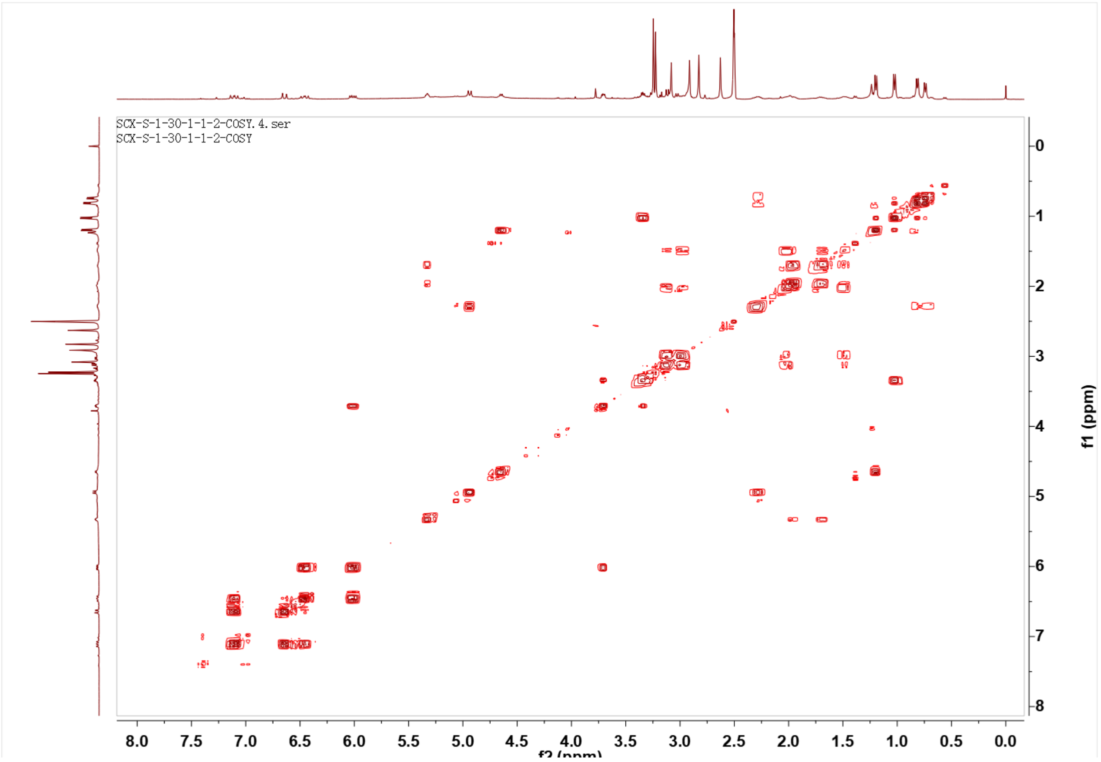

Figure S25. HSQC (400 MHz, DMSO-d_6) spectrum of compound **3**.

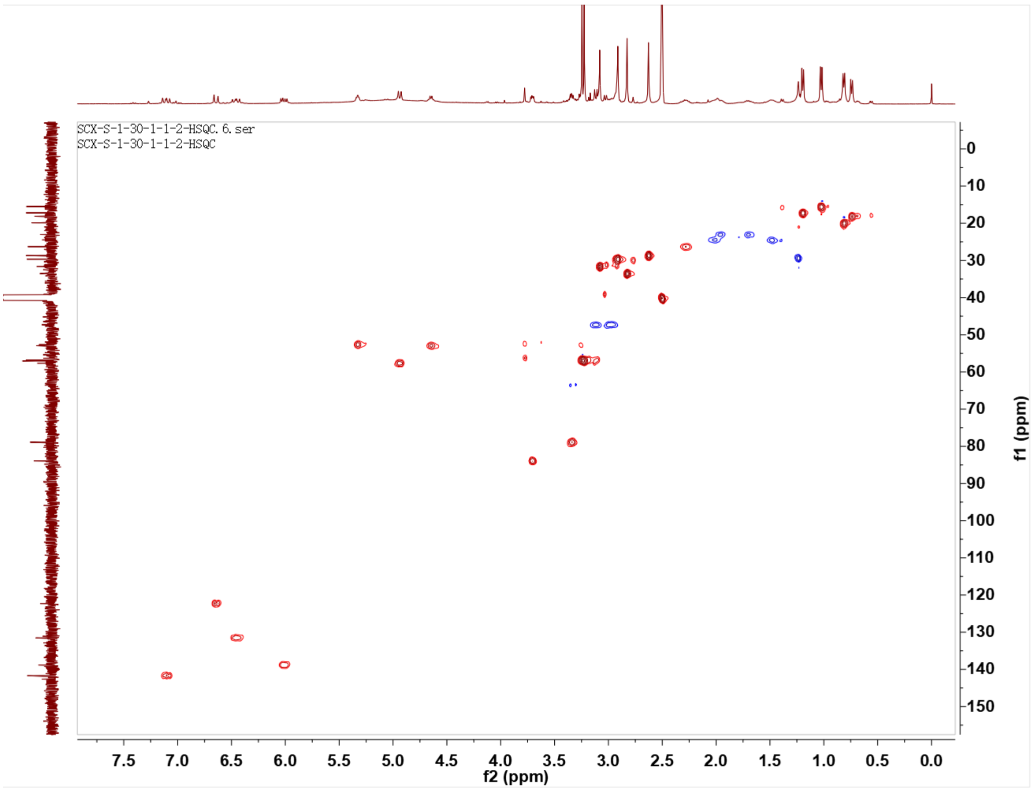

Figure S26. ^{1}H-^{13}C HMBC (400 MHz, DMSO-d_6) spectrum of compound **3**.

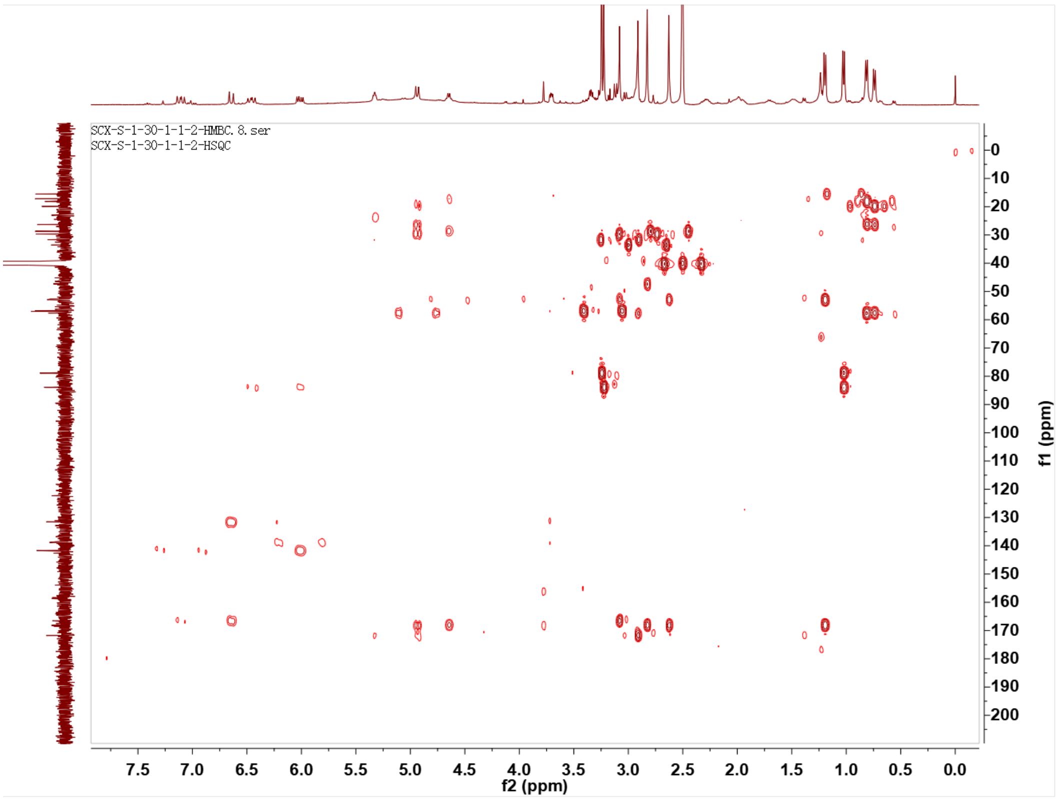

Figure S27. NOESY (400 MHz, DMSO-d_6) spectrum of compound **3**.

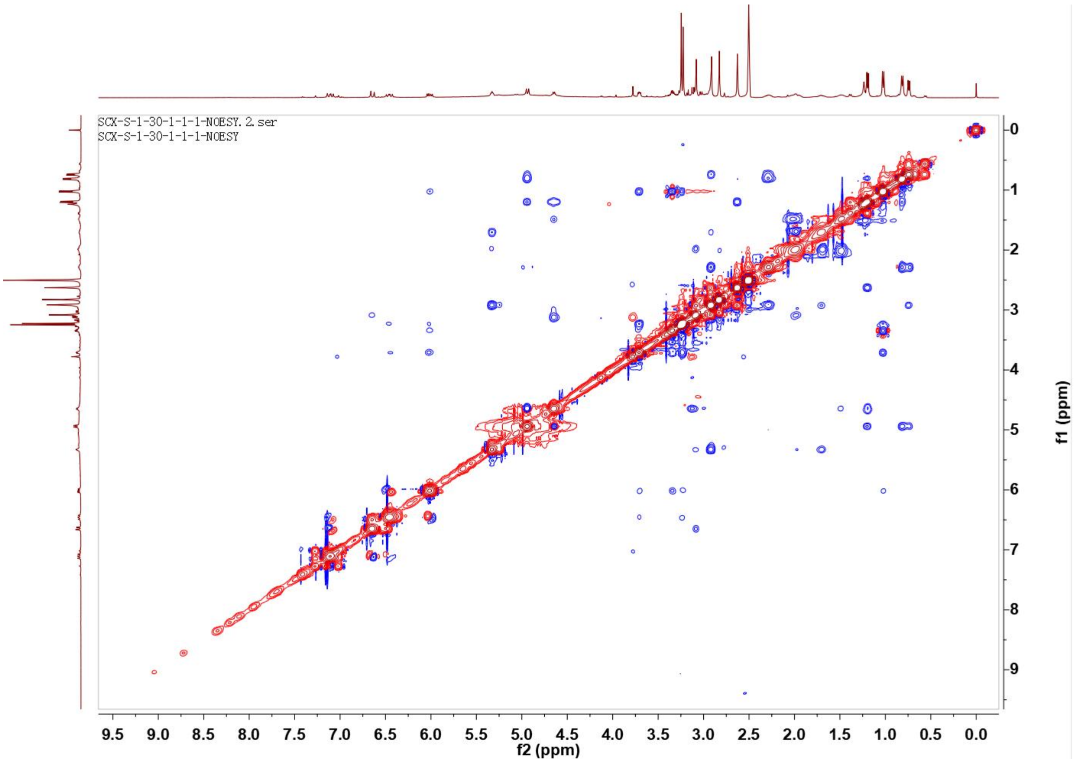

Figure S28. HRESIMS spectrum of compound **3**.

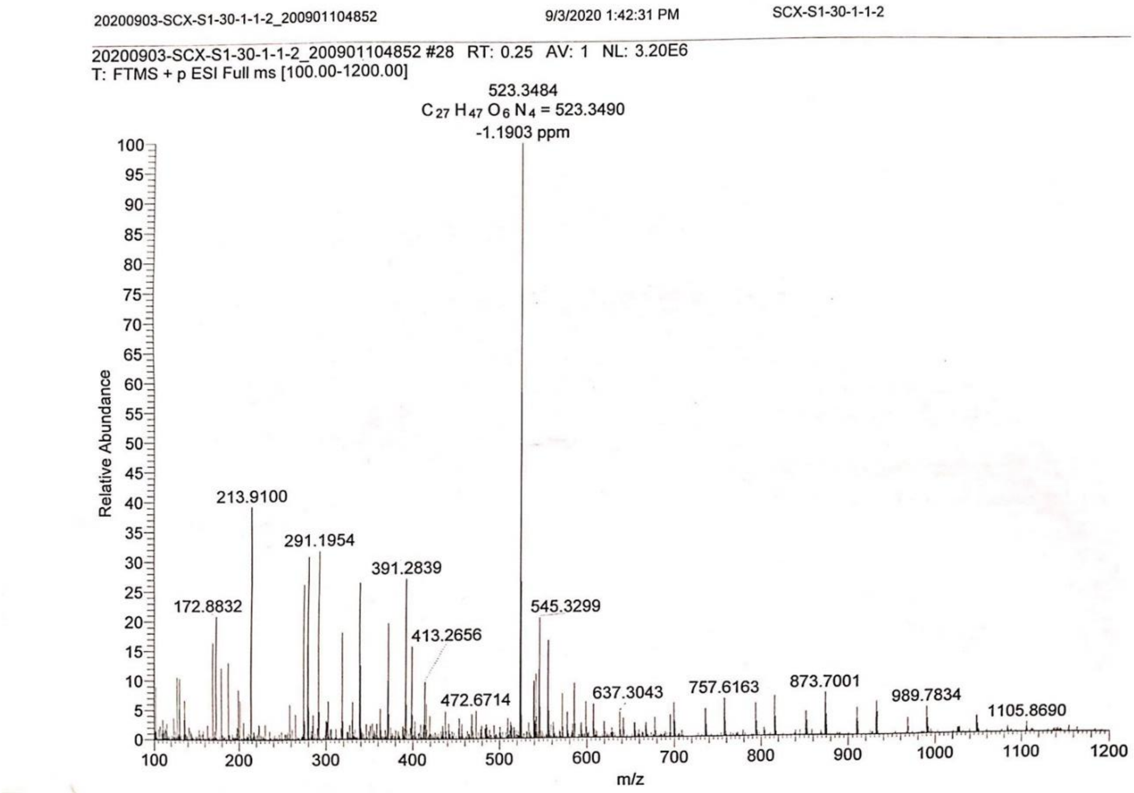

Figure S29. IR spectrum of compound **3**.

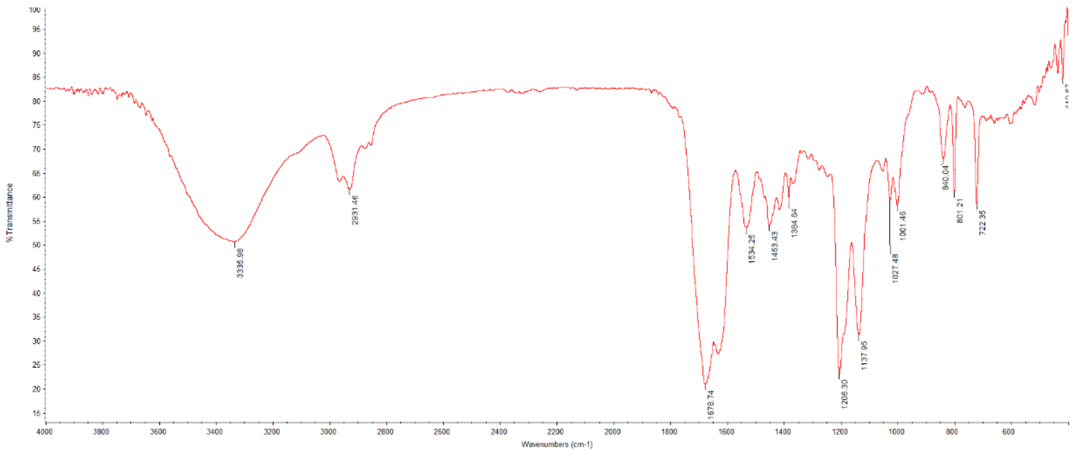

Table S1. ^{1}H NMR (400 MHz) spectroscopic data for compound **4**.

No.	Sclerotiotide L [1]	**4**^{a}	**4**^{b}
2	4.59, q (7.1)	4.59, q (7.1)	4.51, q (7.0)
3	1.51, d (7.1)	1.51, d (7.1)	1.39, d (7.0)
N-CH_3	3.06, s	3.06, s	2.85, s
5	5.11, d (10.5)	5.12, d (10.5)	4.99, d (10.3)
6	2.43, m	2.43, m	2.23, m
7	0.74, d (6.8)	0.74, d (6.8)	0.65, d (6.7)
8	0.92, d (6.3)	0.92, d (6.3)	0.81, d (6.3)
N-CH_3	2.95, s	2.95, s	2.85, s
10	4.98, t (7.2)	4.98, t (7.3)	4.73, t (7.7)
NH/N-CH_3	6.53, d (7.2)	6.54, d (7.3)	8.19, d (7.7)
11	1.60, m	1.60, m	1.67, ov.
	2.39, m	2.39, m	1.97, m
12	1.57, m	1.58, m	1.48, m
	1.66, m	1.66, m	1.66, m
13	3.06, m	3.05, m	2.87, ov.
	3.38, m	3.38, m	3.04, m
NH/N-CH_3	5.68, brs	5.68, brs	7.50, t (6.0)
2'	5.92, d (15.0)	5.92, d (15.0)	6.21, d (15.2)
3'	7.23, dd (15.0, 10.8)	7.24, dd (15.0, 10.8)	7.03, dd (15.1, 11.1)
4'	6.36, dd (15.4, 11.0)	6.36, dd (15.4, 11.0)	6.30, dd (15.3, 11.1)
5'	6.00, dd (15.4, 7.8)	6.00, dd (15.4, 7.8)	5.97, dd (15.3, 7.4)
6'	3.62, dd (7.8, 3.7)	3.62, dd (7.8, 3.7)	3.49, dd (7.4, 4.9)
7'	3.90, dd (6.5, 3.7)	3.90, dd (6.5, 3.7)	3.61, m
8'	1.12, d (6.5)	1.12, d (6.5)	1.02, d (6.3)
9'	3.32, s	3.32, s	3.21, s

^{a} Recorded in $CDCl_3$. ^{b} Recorded in DMSO-d_6.

Figure S30. ^{1}H NMR (400 MHz, $CDCl_3$) spectrum of compound **4**.

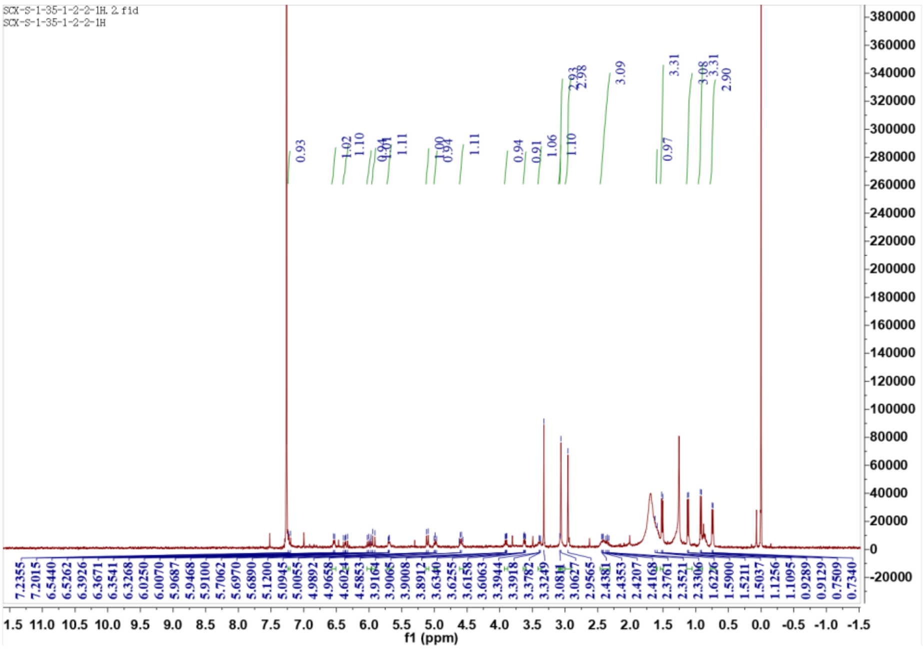

Figure S31. ^{1}H NMR (400 MHz, DMSO-d_6) spectrum of compound **4**.

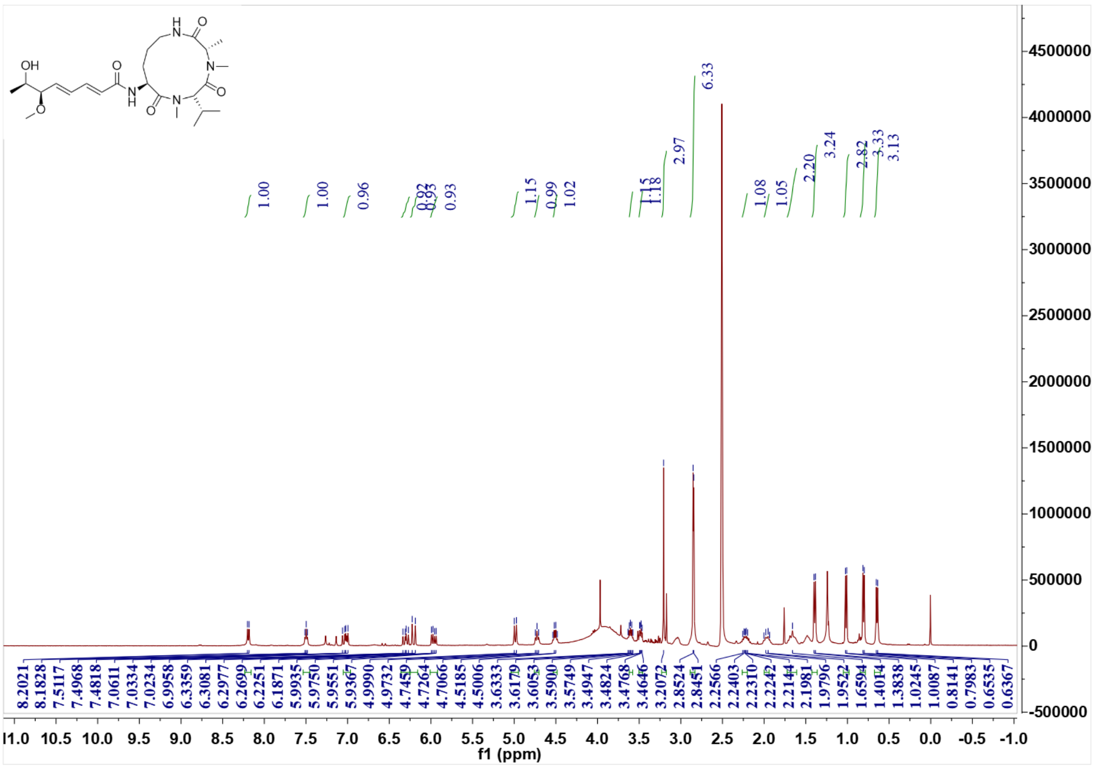

Figure S32. ^{13}C NMR (150 MHz, DMSO-d_6) spectrum of compound **4**.

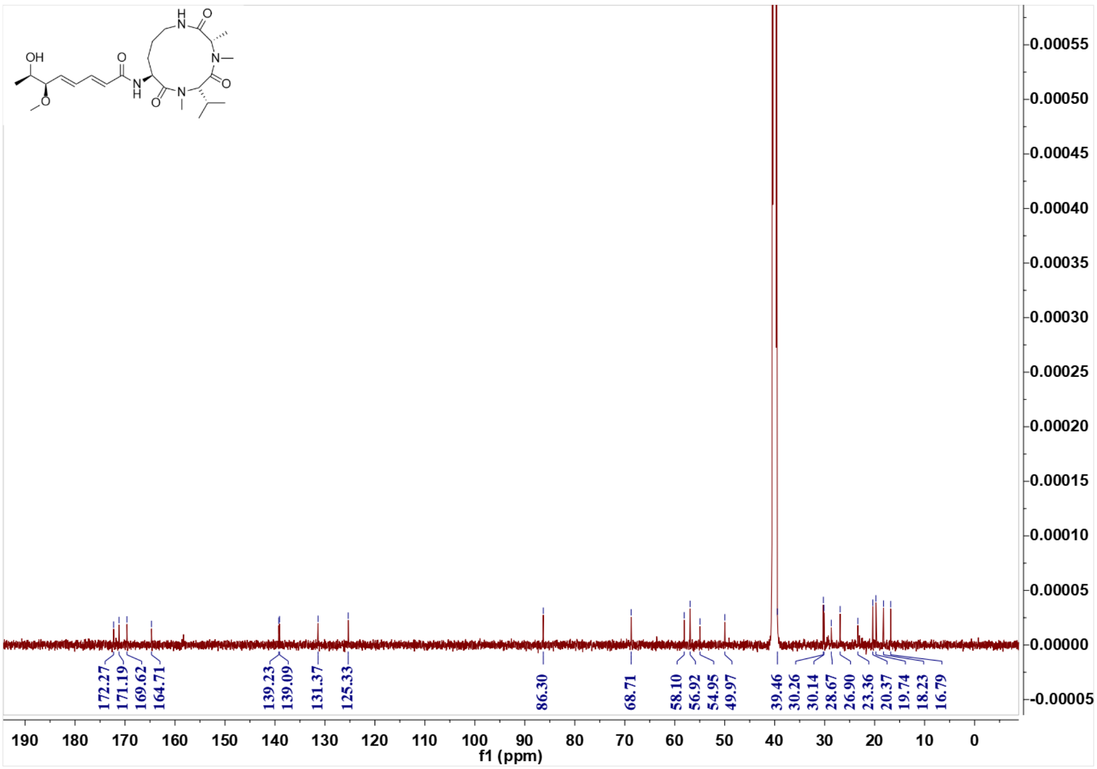

Figure S33. ^{1}H-^{1}H COSY (600 MHz, DMSO-d_6) spectrum of compound **4**.

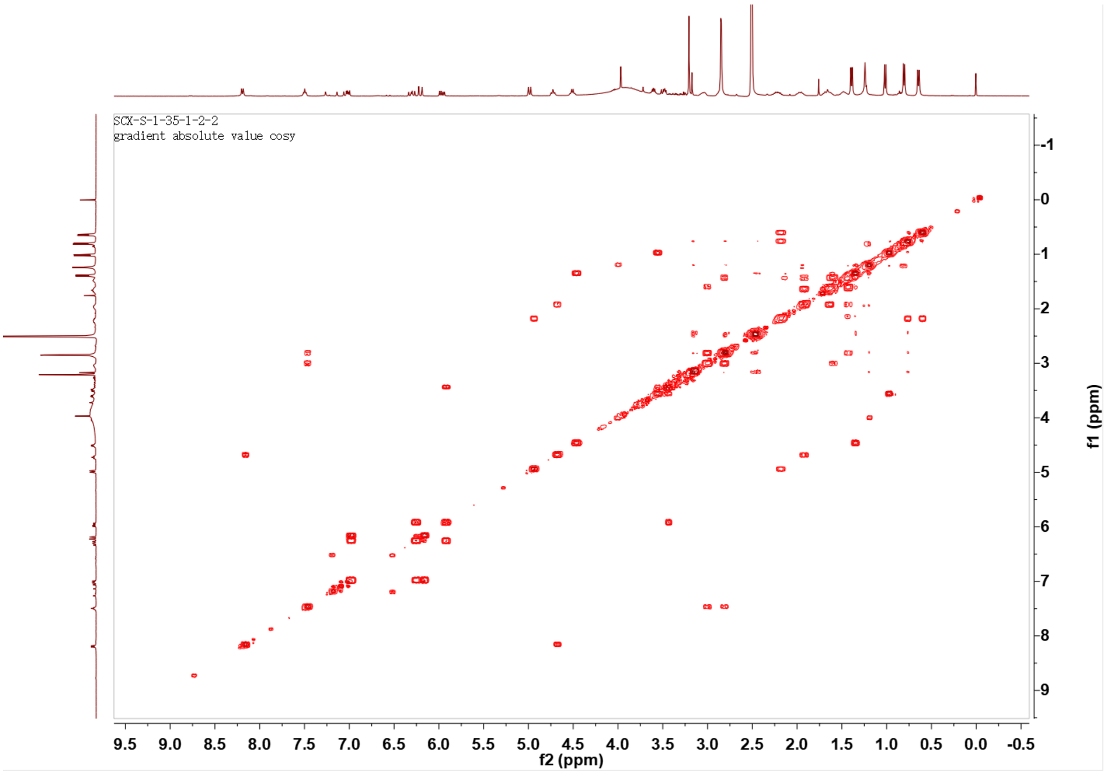

Figure S34. HSQC (600 MHz, DMSO-d_6) spectrum of compound **4**.

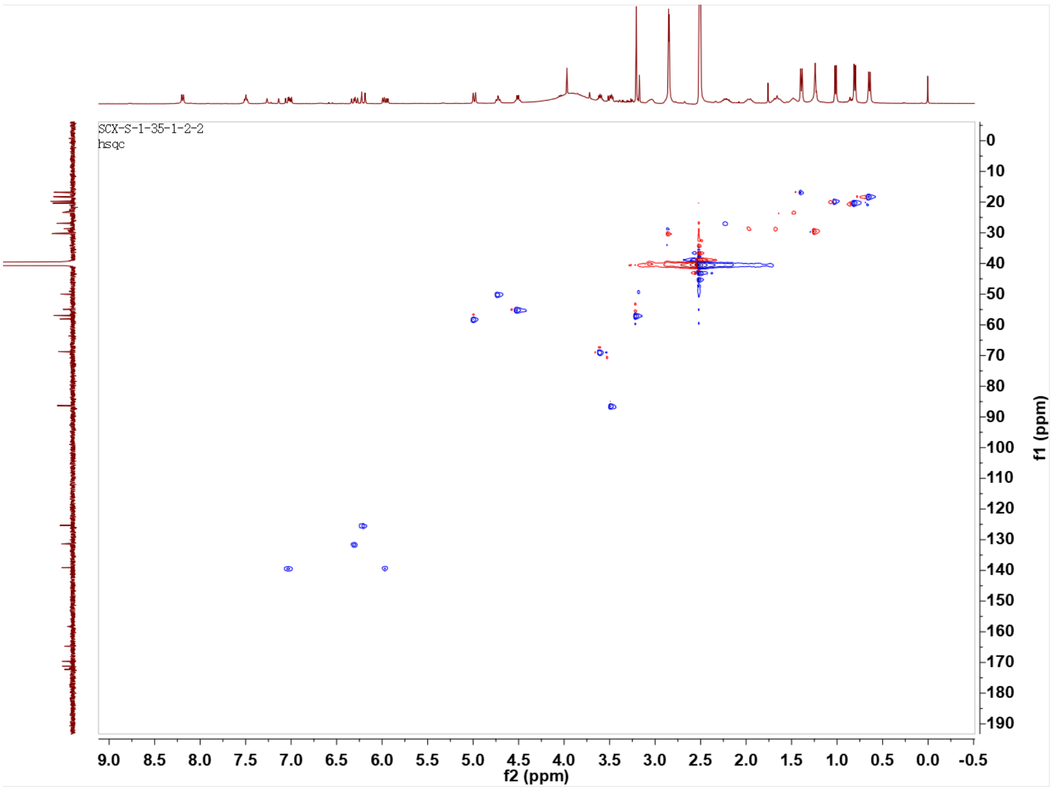

Figure S35. ^{1}H-^{13}C HMBC (600 MHz, DMSO-d_6) spectrum of compound **4**.

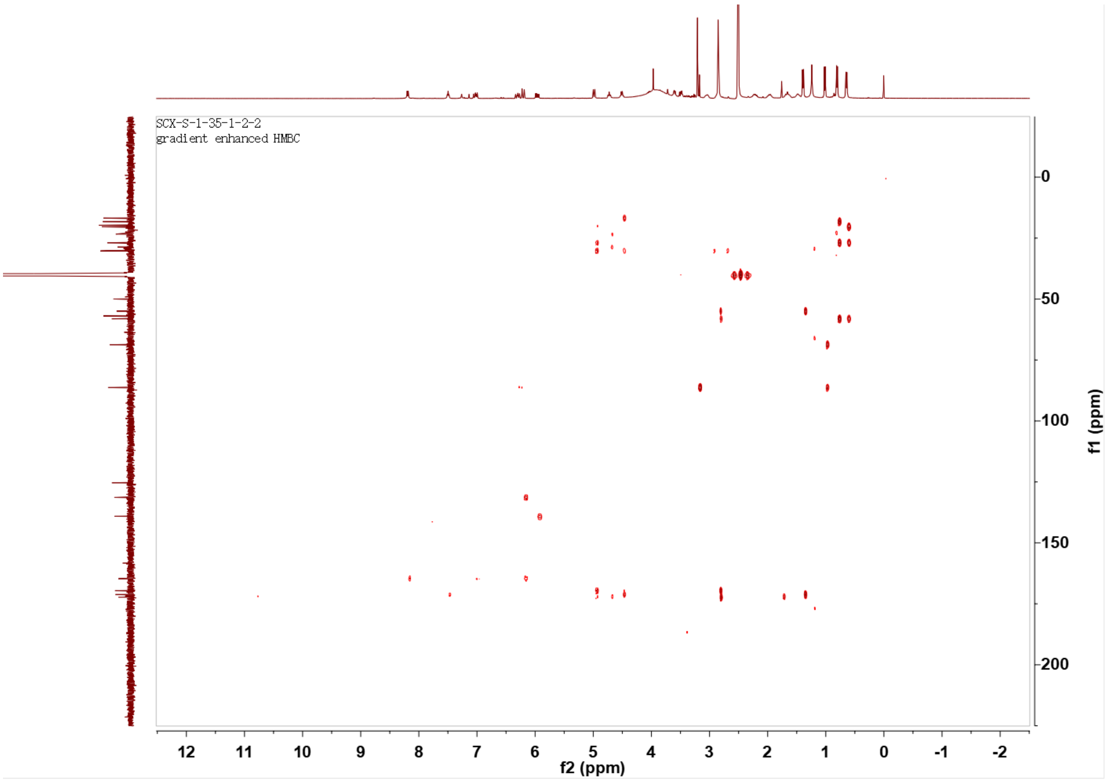

Figure S36. NOESY (600 MHz, DMSO-d_6) spectrum of compound **4**.

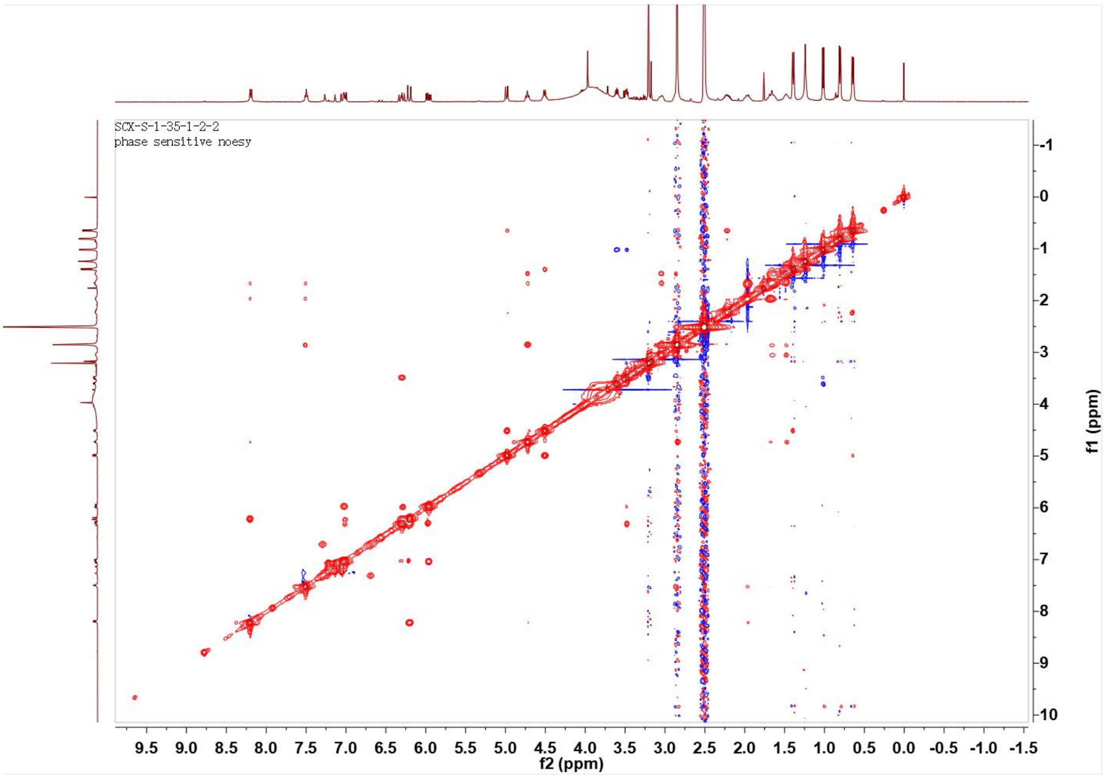

Figure S37. HRESIMS spectrum of compound **4**.

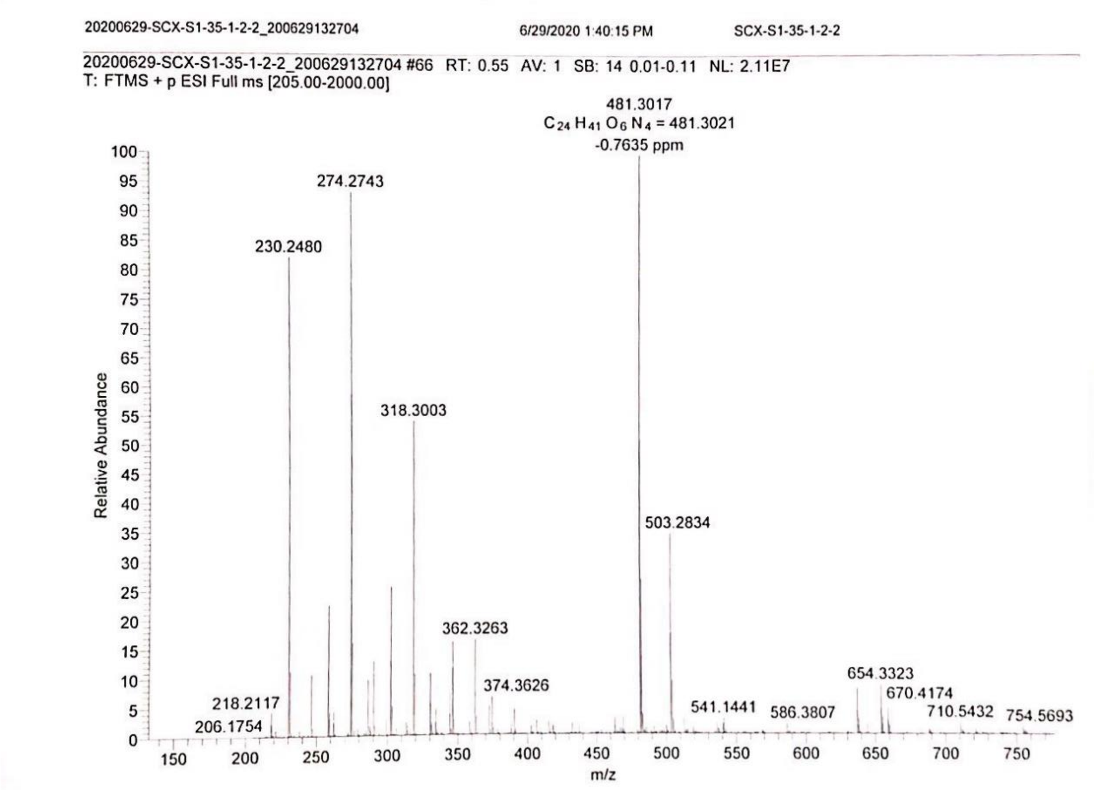

Figure S38. IR spectrum of compound **4**.

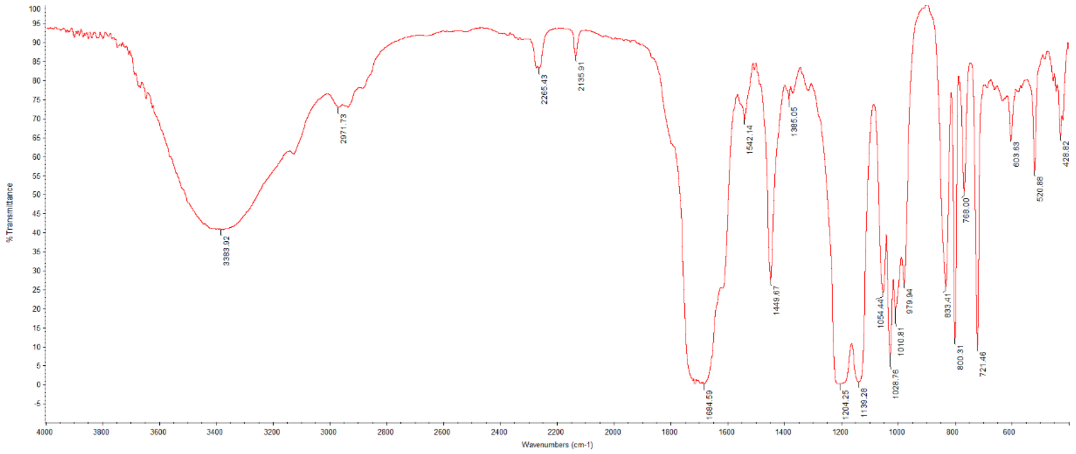

Figure S39. ^{1}H NMR (400 MHz, DMSO-d_6) spectrum of *R*-MPA ester of **4** (**4g**).

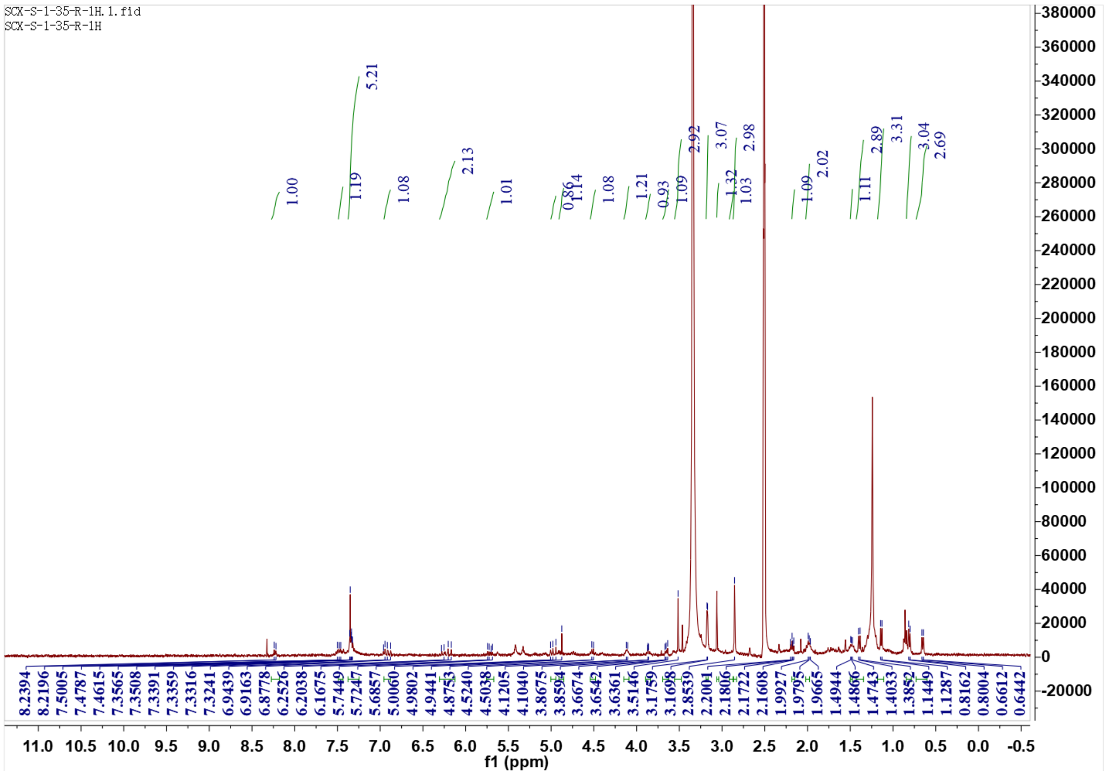

Figure S40. ^{1}H NMR (400 MHz, DMSO-d_6) spectrum of *S*-MPA ester of **4** (**4h**).

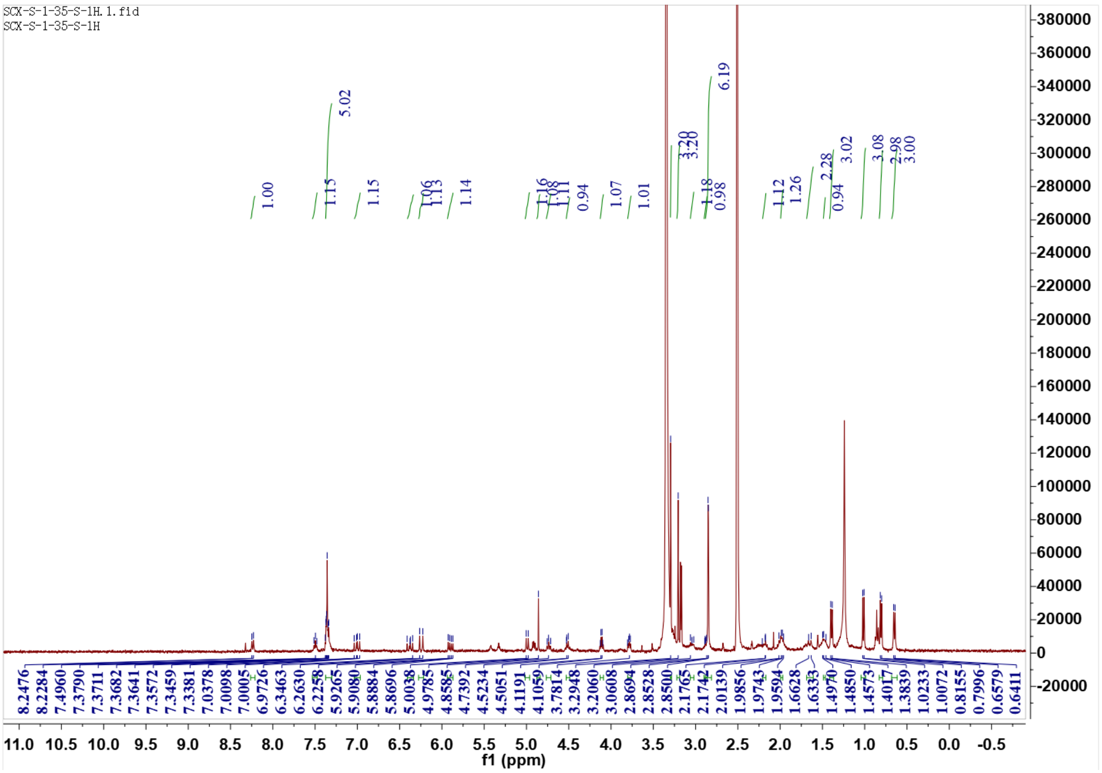

Figure S41. ECD spectra of **3**, **4A**.

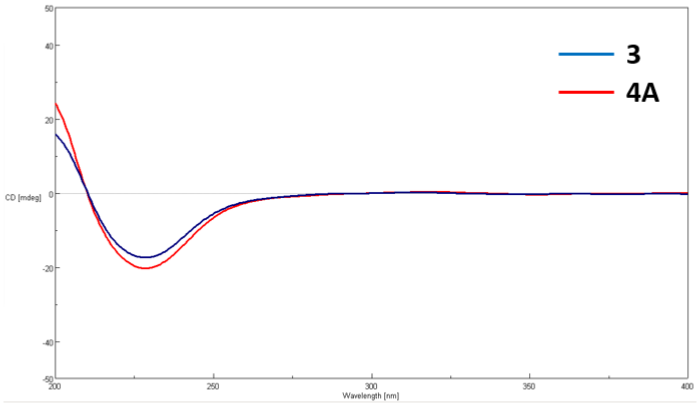

Figure S42. HSQMBC (400 MHz, DMSO-d_6) spectrum of **3**.

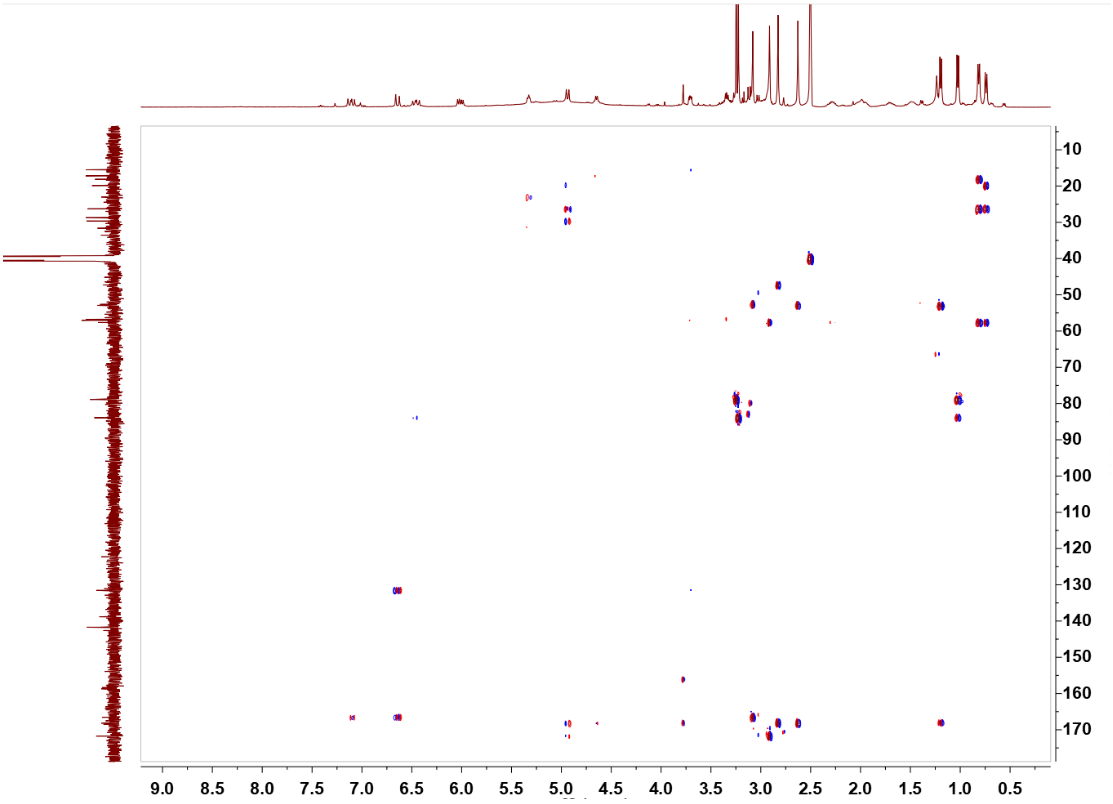

Table S2. ^{1}H NMR parameters of **1-4**.

No.	**1**	**2**	**3**	**4**(DMSO-d_6)	**4**($CDCl_3$)
Data File Name	SCX-S-1-30-3-2	SCX-S-1-50-2-1-4-2	SCX-S-1-30-1-1-2	SCX-S-1-35-1-2-2	SCX-S-1-35-1-2-2
Origin	Varian	Bruker BioSpin GmbH	Bruker BioSpin GmbH	Bruker BioSpin GmbH	Bruker BioSpin GmbH
Instrument	vnmrs	Avance	Avance	Avance	Avance
Solvent	DMSO-d_6	DMSO-d_6	DMSO-d_6	DMSO-d_6	$CDCl_3$
Temperature	298.2	296	297.6	297.6	297.4
Pulse Program	s2pul	zg30	zg30	zg30	zg30
Experiment	1D	1D	1D	1D	1D
Probe	OneProbe	Z163739_0084 (PI HR-400-S1-BBF/ H/ D-5.0-Z SP)	Z163739_0084 (PI HR-400-S1-BBF/ H/ D-5.0-Z SP)	Z163739_0084 (PI HR-400-S1-BBF/ H/ D-5.0-Z SP)	Z163739_0084 (PI HR-400-S1-BBF/ H/ D-5.0-Z SP)
Number of Scans	32	16	16	32	16
Receiver Gain	54	101	101	101	101

Relaxation Delay	1	1	1	1	1
Pulse duration	3.75	8	8	8	8
Acquisition Time	2.0447	3.9977	3.9977	3.9977	3.9977
Acquisition Date	2019-08-09T13:29:52	2019-12-07T12:13:17	2020-08-24T18:09:42	2019-09-26T23:42:25	2020-07-27T21:35:02
Modification Date	2019-08-09T13:31:34	2019-12-07T12:12:56	2020-08-24T18:09:52	2019-09-26T23:42:08	2020-07-27T21:34:12
Spectrometer Frequency	499.82	400.13	400.13	400.13	400.13
Spectral Width	8012.8	8196.7	8196.7	8196.7	8196.7
Lowest Frequency	-1007.5	-1629.5	-1629.2	-1627.6	-1636.1
Nucleus	1H	1H	1H	1H	1H
Acquired Size	16384	32768	32768	32768	32768
Spectral Size	65536	65536	65536	65536	65536

Table S3. ^{13}C NMR parameters of **1-4**.

No.	**1**	**2**	**3**	**4**
Topic	SCX-S-1-30-3-2	SCX-S-1-50-2-1-4-2	SCX-S-1-30-1-1-2	SCX-S-1-35-1-2-2
Origin	Varian	JEOL	Bruker BioSpin GmbH	JEOL
Instrument	vnmrs	ECA	Avance	ECA
Solvent	DMSO-d_6	DMSO-d_6	DMSO-d_6	DMSO-d_6
Temperature	298.2	295.3	298.3	295.3
Pulse Program	s2pul	carbon.jxp	zgpg30	carbon.jxp
Experiment	1D	1D	1D	1D
Probe	OneProbe	2756	Z163739_0084 (PI HR-400-S1-BBF/ H/ D-5.0-Z SP)	2756
Number of Scans	12804	7714	2500	5000
Receiver Gain	30	36	21.2	36
Relaxation Delay	1	2	2	2
Pulse duration	4.75	3.2083	8	3.2083
Acquisition Time	1.0486	0.6921	1.3763	0.6921
Acquisition Date	2019-08-22T03:25:58	2019-12-23T01:25:30	2020-08-26T02:59:44	2019-10-05T08:02:37

Modification Date	2019-08-22T10:43:56	2019-12-24T10:52:49	2020-08-26T02:59:46	2019-10-05T11:47:13
Spectrometer Frequency	125.69	150.92	100.62	150.92
Spectral Width	31250	37876.8	23809.5	37878.8
Lowest Frequency	-1800.4	-3846.3	-1843.5	-3847.3
Nucleus	13C	Carbon13	13C	Carbon13
Acquired Size	32768	32768	32768	32768
Spectral Size	65536	26214	65536	52430

Reference

[1] Liu, J.; Gu, B.; Yang, L.; Yang, F.; Lin, H. New Anti-inflammatory cyclopeptides from a sponge-derived fungus *Aspergillus violaceofuscus*. *Front. Chem.* **2018**, *6*, 226-233. DOI: 10.3389/fchem.2018.00226.
